# POLD4 Promotes Glioma Cell Proliferation and Suppressive Immune Microenvironment: A Pan-Cancer Analysis Integrated with Experimental Validation

**DOI:** 10.3390/ijms241813919

**Published:** 2023-09-10

**Authors:** Cheng Jiang, Fei Fan, Weiming Xu, Xiaobing Jiang

**Affiliations:** Department of Neurosurgery, Union Hospital, Tongji Medical College, Huazhong University of Science and Technology, Wuhan 430000, Chinaxuweiming9@126.com (W.X.)

**Keywords:** DNA polymerase delta subunit 4 (POLD4), proliferation, immunosuppressive microenvironment, immunomarker, pan-cancer, gliomas

## Abstract

POLD4 plays a crucial part in the complex machinery of DNA replication and repair as a vital component of the DNA polymerase delta complex. In this research, we obtained original information from various publicly available databases. Using a blend of R programming and internet resources, we initiated an extensive examination into the correlation between POLD4 expression and the various elements of cancers. In addition, we performed knockdown experiments in glioma cell lines to authenticate its significant impact. We discovered that POLD4 is upregulated in various malignant tumors, demonstrating a significant correlation with poor patient survival prognosis. Using function analysis, it was uncovered that POLD4 exhibited intricate associations with signaling pathways spanning multiple tumor types. Subsequent investigations unveiled the close association of POLD4 with the immune microenvironment and the effectiveness of immunotherapy. Drugs like trametinib, saracatinib, and dasatinib may be used in patients with high POLD4. Using experimental analysis, we further confirmed the overexpression of POLD4 in gliomas, as well as its correlation with glioma recurrence, proliferation, and the suppressive immune microenvironment. Our research findings indicate that the expression pattern of POLD4 not only serves as a robust indicator of prognosis in cancer patients but also holds promising potential as a new focus for treatment.

## 1. Introduction

Cancer is a devastating disease characterized by uncontrolled cell growth and proliferation, posing a significant global health burden. Despite remarkable strides in medical technology and the expanding array of treatment alternatives for cancer, a significant proportion of patients receive diagnoses at an advanced stage, rendering conventional therapeutic strategies impractical and culminating in fatal outcomes associated with the disease [1,2]. Recognizing the limitations of existing treatment methods, there is a pressing requirement to encourage the development of fresh targets for cancer therapy, intended to enhance precise diagnosis and successful treatment approaches [3].

Up until now, although it has been suggested that abnormalities in DNA replication contribute to the progression of cancer, there has been limited identification of core DNA replication proteins as commonly altered entities in cancer [4,5]. We conducted extensive bioinformatics analyses, screening for DNA replication genes of significant biological importance across different cancer types. POLD4 stood out due to its intriguing expression patterns, correlation with prognosis, and functional enrichment in multiple cancer types, which piqued our interest. The essential DNA replication complex known as pol δ in eukaryotes is made up of four distinct subunits: POLD1, POLD2, POLD3, and POLD4. With its exonuclease activity, this enzyme significantly enhances proofreading capabilities, crucially upholding the precision of DNA replication [6]. Additionally, it aids in the restoration of DNA damage resulting from mutagen exposure [7]. Over the past decade, an increasing body of research established a connection between mutations in Pol δ and various human pathologies [8]. Playing a fundamental role in DNA replication and repair processes, POLD4 serves as the smallest subunit of Pol δ [9]. It acts as a crucial mediator of faithful DNA synthesis and maintenance of genomic stability. Currently, research on POLD4 in cancer is primarily focused on lung cancer. Research has indicated that a significant tobacco carcinogen, the benzopyrene analogue 4NQO, downregulated the expression level of the POLD4 gene, resulting in a decreased nucleotide excision repair and further leading to genomic instability, ultimately elevating the risk of lung cancer formation [10]. Huang and colleagues discovered that reducing POLD4 levels in lung cancer cells not only delays the progression via the G1-S cell cycle transition but also results in heightened genomic instability [11]. Subsequently, the team conducted additional research and found that the reduction in POLD4 in Calu6 cells leads to G1-S blockage by inhibiting the Akt-Skp2-p27 pathway [12]. The reduction in POLD4 mediated by shRNA resulted in a significant decrease in the colony formation activity of Calu6, ACC-LC-319, and PC-10 cells [13]. Additionally, evidence indicates that POLD4 may serve as a promising novel target for lung carcinoma, given its selective impact on cancer cells without affecting normal cells [9]. However, the exploration of POLD4 in other cancer types and the in-depth mechanisms of its actions remain limited, and its complete function has yet to be fully clarified.

Integrated bioinformatics and public databases are indispensable in cancer research, providing a centralized platform for aggregating diverse datasets. These resources encompass genomic, gene expression, clinical, and drug response data, enabling a holistic comprehension of cancer biology. Additionally, they promote global collaboration, empowering researchers to share and analyze data, and facilitating meta-analyses, pattern identification, and biomarker discovery. These databases offer critical analysis tools, from basic sequence alignment to advanced machine learning, aiding in data interpretation. Moreover, they drive personalized medicine by identifying genetic factors influencing cancer predisposition, prognosis, and treatment response.

By integrating computational analysis with experimental data, our goal is to provide valuable insights into the potential therapeutic targeting of POLD4 and the modulation of the immune microenvironment in cancers.

## 2. Results

### 2.1. POLD4 Is Upregulated in Pan-Cancer Tissues

**Figure 1:**
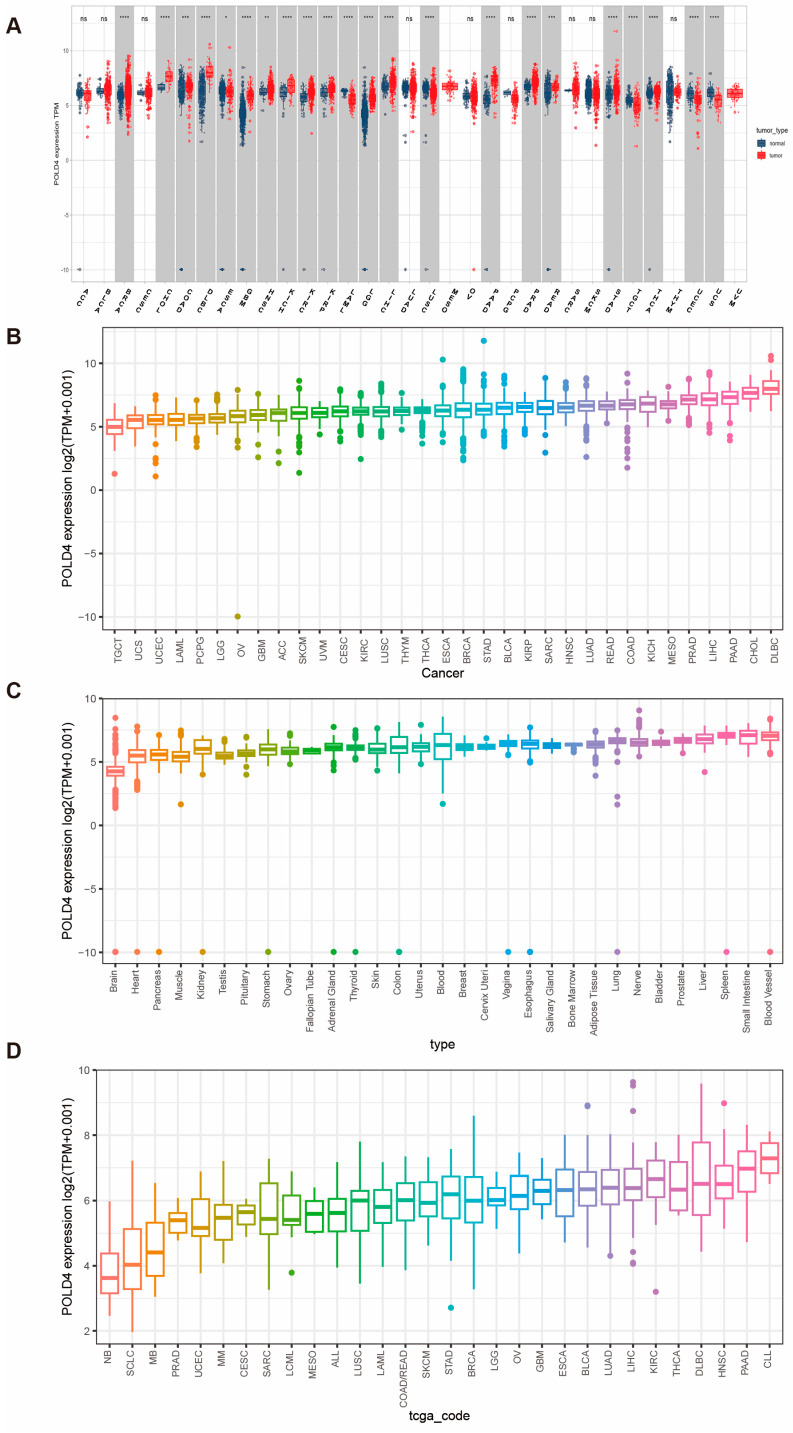
POLD4 expression levels in tissues and cell lines. (**A**) Differential POLD4 expressions between tumor tissues and normal tissues were analyzed using the TCGA and GTEx databases. (**B**) POLD4 expression box plot in tumor tissues from TCGA, showing increasing expression from left to right. (**C**) POLD4 expression box plot in normal tissues from GTEx, showing increasing expression from left to right. (**D**) POLD4 expression box plot in tumor cell lines from CCLE, showing increasing expression from left to right. ns. *p* > 0.05, * *p* < 0.05, ** *p* < 0.01, *** *p* < 0.001, and **** *p* < 0.0001.

GTEx, or Genotype-Tissue Expression, differs from TCGA in that it primarily focuses on sequencing data from normal individuals. TCGA predominantly collects cancer-related data, with relatively limited normal tissue samples. This scarcity of normal samples may lead to less precise results in differential expression analysis. In such instances, incorporating GTEx data enhances the accuracy of the analysis. By integrating the GTEx and TCGA databases, we conducted an analysis to examine the variation in POLD4 expression across 33 distinct cancer types. A comprehensive inventory of these cancer types, along with their corresponding abbreviations, can be accessed in Table S1. In the TCGA cohort, POLD4 displayed distinct expression patterns in various cancer types. Heightened expression levels were evident in BRCA, CHOL, COAD, DLBC, ESCA, GBM, HNSC, KICH, KIRC, LGG, LIHC, LUSC, PAAD, PRAD, READ, STAD, and THCA. Conversely, diminished expression levels were observed in LAML, TGCT, UCEC, and UCS, as depicted in Figure 1A. Figure 1B,C, respectively, depict the expression distribution of POLD4 in different cancer types and tissues, arranged in ascending order. Based on data extracted from the CCLE database, it was discovered that the HNSC, PAAD, and CLL cells exhibited the most elevated levels of POLD4 expression (Figure 1D). Supplements 1a-k display the outcomes of expression analysis for POLD4 in various pairs of tumor and adjacent normal tissues derived only from the TCGA dataset. In this section, POLD4 exhibited upregulation in ESCA, BRCA, CHOL, HNSC, KICH, KIRC, KIRP, LIHC, PRAD, STAD, and THCA (Figure S1A–K). However, COAD, LUSC, and READ showed downregulated expression compared to adjacent normal tissues (Figure S1L–N). The differential expression of POLD4 across various cancer types underscores its potential as a diagnostic and prognostic marker. Elevated POLD4 expression may serve as an indicator of disease progression or severity in specific cancer types, which could aid in patient stratification and treatment decisions. Secondly, the distinct expression patterns in different cancer types suggest that POLD4 may play a context-specific role in oncogenesis. Understanding the mechanistic underpinnings of these variations could provide insights into the molecular basis of cancer and potentially open avenues for targeted therapies.

### 2.2. POLD4 and Gene Mutation Landscape

The use of cBioPortal to analyze genetic alterations in POLD4 demonstrated that the gene exhibited the highest alteration frequency (>6%) in patients diagnosed with esophageal adenocarcinoma. Notably, the most prevalent alteration observed was characterized as “amplification.” (Figure S2). Following that, we conducted an analysis exploring the connection between POLD4 expression and relative linear copy number values. Our findings revealed a positive correlation between POLD4 expression and copy number alterations (CNA) in various tumor types, with the exception of CHOL, MESO, LAML, GBM, KIRP, UCEC, KIRC, PRAD, UVM, and ACC (Figure S3A). The correlation between POLD4 expression and copy number values in GBM and LGG is shown separately in Figure S3B,C. In addition, we present waterfall plots showing the mutation profiles of the high POLD4 group and the low POLD4 group in both GBM and LGG (Figure S4).

### 2.3. Prognostic Significance of POLD4

**Figure 2:**
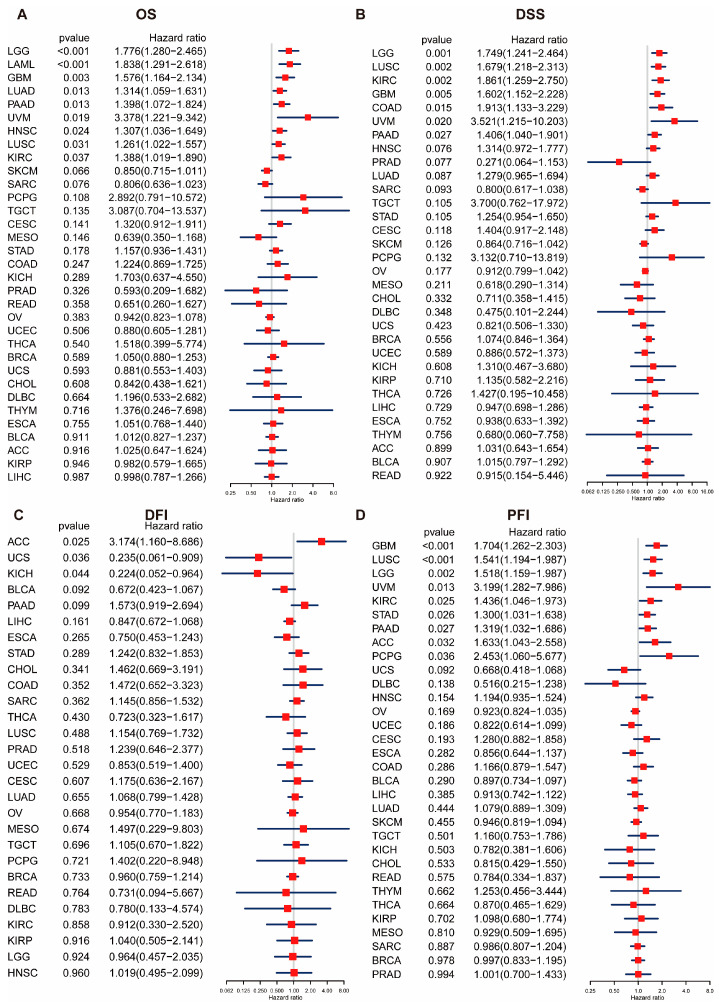
Prognostic value of POLD4 in pan-cancer analyzed using Univariate Cox regression analysis. (**A**) Relationship between POLD4 expression and overall survival (OS) in pan-cancer. (**B**) Relationship between POLD4 expression and disease-specific survival (DSS) in pan-cancer. (**C**) Relationship between POLD4 expression and disease-free interval (DFI) in pan-cancer. (**D**) Relationship between POLD4 expression and progression-free interval (PFI) in pan-cancer.

The Cox proportional hazards model was employed to investigate the correlation between POLD4 levels and the survival outcomes of patients, such as overall survival (OS), disease-specific survival (DSS), disease-free interval (DFI), and progression-free interval (PFI). The findings demonstrated a significant association between elevated POLD4 levels and poorer OS in various cancer types, including LGG, LAML, GBM, LUAD, PAAD, and UVM (Figure 2A). In terms of DSS, a notable correlation was observed between elevated expression of POLD4 and poorer clinical outcomes in LGG, LUSC, KIRC, GBM, COAD, UVM, and PAAD (Figure 2B). Furthermore, a remarkable correlation was observed between the elevated levels of POLD4 and markedly reduced DFI in patients with ACC (Figure 2C). In GBM, LUSC, LGG, UVM, KIRC, STAD, PAAD, ACC, and PCPG, the correlation between high POLD4 expression and reduced PFI was found to be statistically significant (Figure 2D). POLD4 exerts a significant impact on the survival of gliomas, including both LGG and GBM. Therefore, in subsequent research, we placed particular emphasis on investigating the effects of POLD4 on gliomas. Moreover, the survival durations in GBM, COAD, HNSC, KIRC, LAML, LGG, LUAD, LUSC, PESO, PAAD, PCPG, SARC, STAD, TGCT, UVM, and SKCM were significantly reduced with an elevation in POLD4 expression, as evidenced by the Kaplan–Meier curves (Figure S5). The link between POLD4 expression and increased or decreased risk can be attributed to its role in DNA replication and repair processes. The high expression of POLD4 has been shown to enhance DNA replication fidelity, reducing the risk of accumulating mutations that could lead to cancer initiation or progression. Conversely, elevated POLD4 expression might also indicate increased cellular proliferation, potentially contributing to tumor growth and progression. In some cases, this enhanced proliferation could lead to more aggressive forms of cancer, thereby correlating with decreased survival rates.

### 2.4. POLD4 Plays Multiple Oncogenic Roles in Gliomas

**Figure 3:**
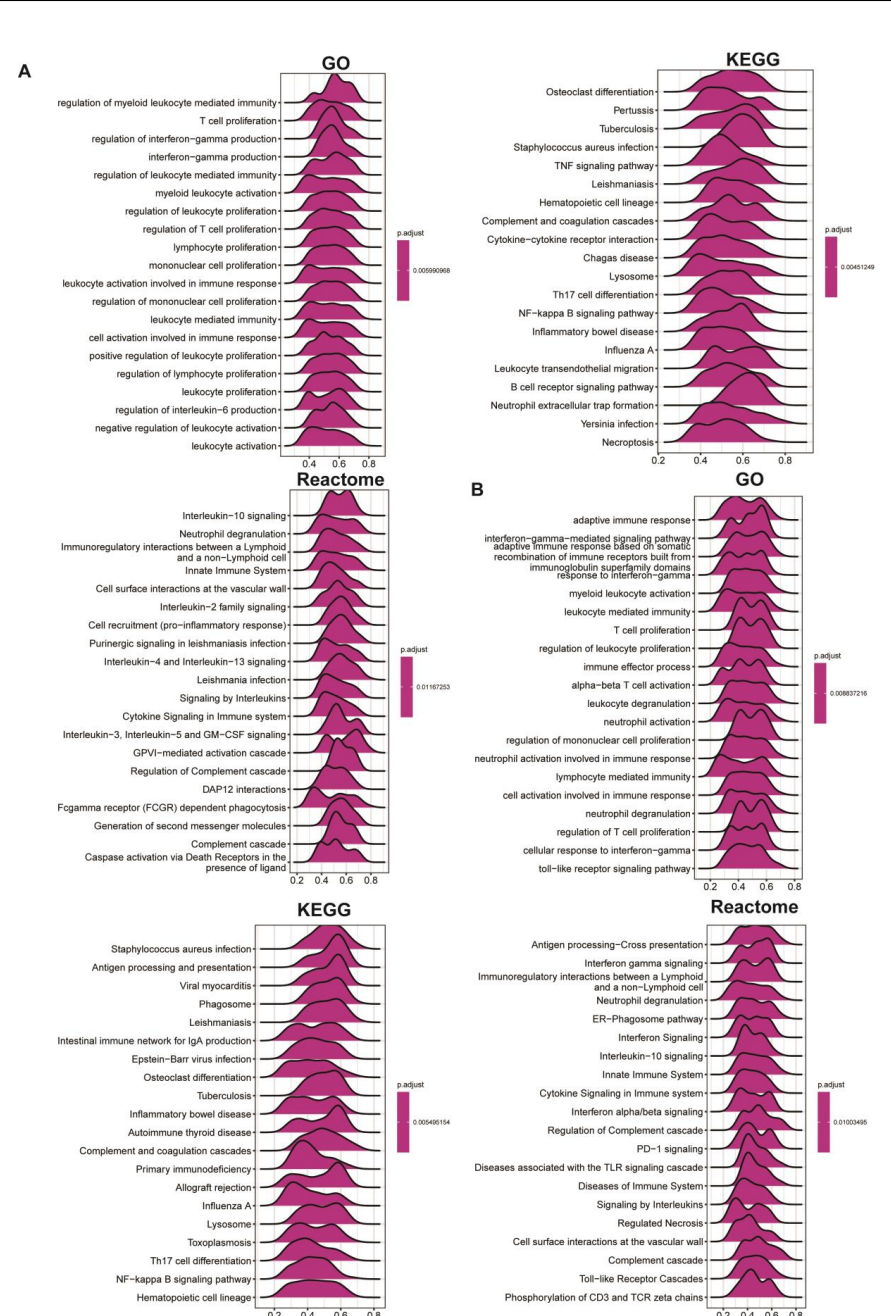
GSEA analysis for POLD4-related genes in GBM and LGG. (**A**) Top 20 results of GSEA analysis for POLD4-related genes in GBM with GO, KEGG, and Reactome annotations. (**B**) Top 20 results of GSEA analysis for POLD4-related genes in LGG with GO, KEGG, and Reactome annotations.

To analyze the oncogenic roles of POLD4 in gliomas, we conducted an analysis of the expression of POLD4 and its correlation with other genes in both GBM and LGG cases. The expression of the top 50 genes positively and negatively correlated with POLD4 was displayed using heatmaps. In GBM, ARPC1B displayed the strongest positive correlation with POLD4, whereas ZNF519 demonstrated the most pronounced negative correlation with POLD4 (Figure S6A). In LGG, GRN exhibited the highest positive correlation with POLD4, whereas FAM81A showcased the most substantial negative correlation with POLD4 (Figure S6B). Next, we selected the top genes that were most significantly correlated with POLD4 to form a gene set for enrichment analysis, aiming to predict the functional pathways of this gene. This process was performed separately in GBM and LGG. The GSEA results for POLD4-related genes in GBM and LGG are depicted in Figure 3A,B, respectively, highlighting the top 20 outcomes for Gene Ontology (GO), Kyoto Encyclopedia of Genes and Genomes (KEGG), and Reactome. In these results, we can observe a significant abundance of enrichment outcomes related to immunity, such as regulation of myeloid leukocyte-mediated immunity, T cell proliferation, regulation of leukocyte-mediated immunity, and the cell activation involved in immune responses. DNA replication and repair processes are critical for maintaining genomic integrity. The dysregulation of these processes can lead to DNA damage, which, in turn, activates the DNA damage response pathway. Several DDR components have been shown to influence immune responses. For example, the activation of DDR can lead to the upregulation of immune checkpoint molecules like PD-L1, which can suppress antitumor immune responses. Therefore, POLD4 may indirectly regulate tumor immunity by influencing DNA replication and repair.

**Figure 4:**
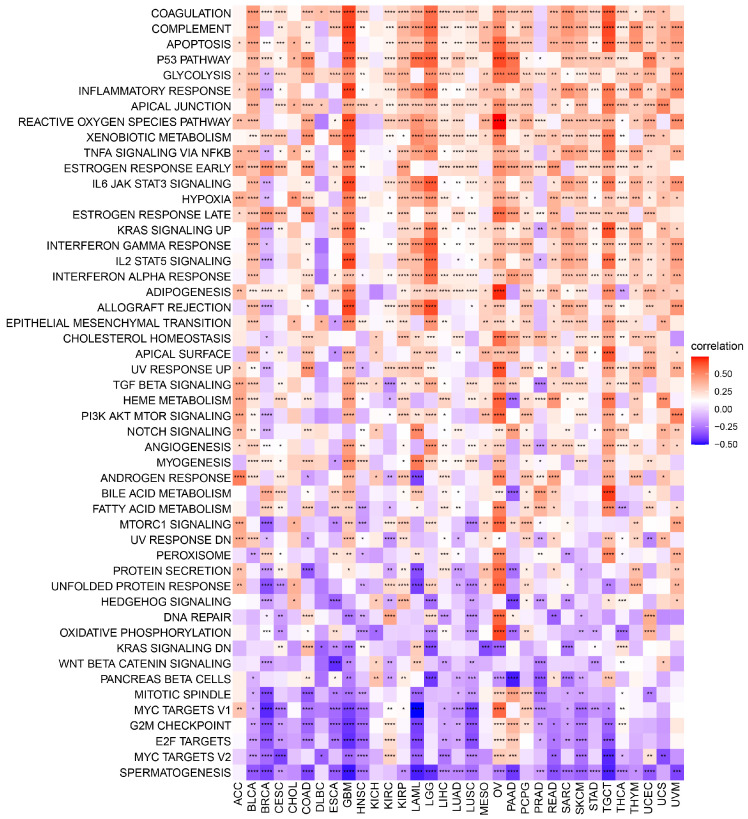
GSVA analysis for POLD4 in pan-cancer. * *p* < 0.05, ** *p* < 0.01, *** *p* < 0.001, and **** *p* < 0.0001.

Following that, a GSVA analysis was performed to investigate the potential impact of POLD4 expression on 50 HALLMARK pathways. The graphical representation in Figure 4 illustrates the correlation between pan-cancer POLD4 expression and GSVA pathways. In our discovery, we observed the involvement of POLD4 in numerous pathways that contribute to cancer progression and immune regulation. These pathways encompass Glycolysis, TGF Beta signaling, PI3K/AKT/MTOR signaling, as well as Interferon Alpha and Interferon Gamma Responses across various cancer types. Specifically, we observed some pathways related to the tumor’s response to stress, such as Apoptosis, P53 pathway, Reactive Oxygen Species pathway, and Hypoxia. The enrichment analysis results of POLD4 in GBM and LGG using GSVA are presented separately based on the correlation coefficients. The pathways exhibiting the highest correlation with POLD4 expression in GBM include Inflammatory Response, Complement, Allograft Rejection, Coagulation, IL6/JAK/STAT3 Signaling, IL2/STAT5 Signaling, Apoptosis, and Reactive Oxygen Species pathway (Figure S7). As for LGG, the pathways showing the strongest correlation with POLD4 expression comprise Allograft Rejection, IL6/JAK/STAT3 signaling, Interferon Gamma Response, P53 pathway, IL2/STAT5 signaling, Inflammatory Response, Complement, and Coagulation (Figure S8). POLD4’s enrichment in these pathways suggests its involvement in various aspects of cancer progression and immune regulation. To fully understand the mechanisms, further experimental validation, and functional studies are needed to determine how POLD4 interacts with specific components within these pathways and how these interactions contribute to its role in cancer biology and the immune microenvironment.

**Figure 5:**
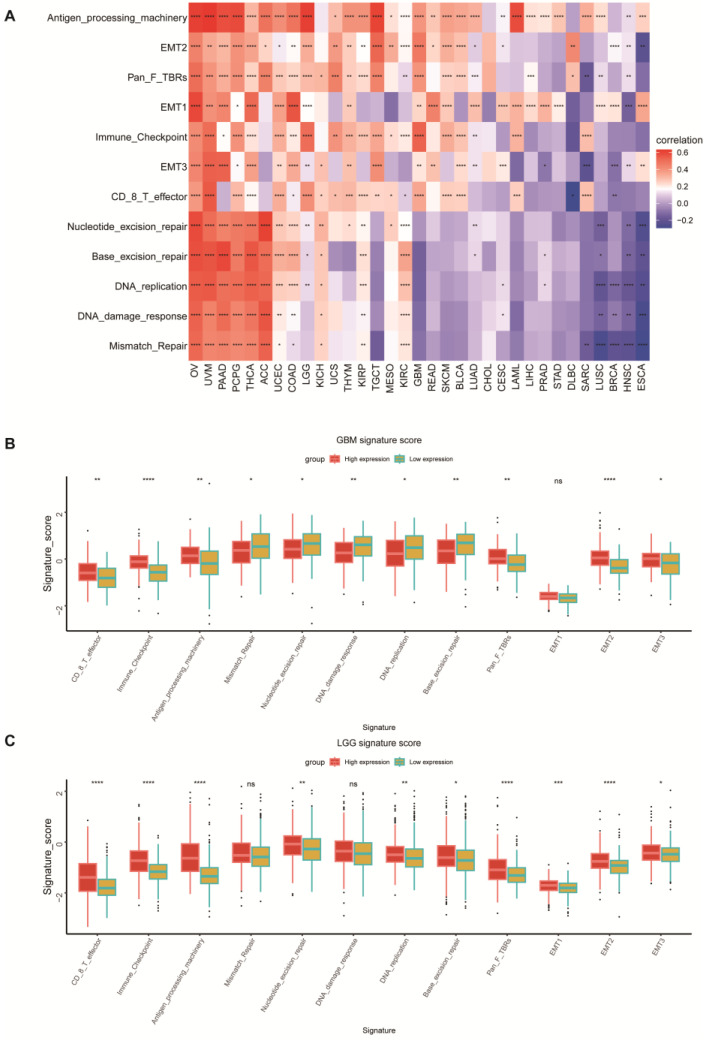
Correlation between POLD4 expression and reported signatures. (**A**) Correlation between POLD4 expression and reported signatures in pan-cancer. (**B**) Correlation between POLD4 expression and reported signatures in GBM. (**C**) Correlation between POLD4 expression and reported signatures in LGG. ns. *p* > 0.05, * *p* < 0.05, ** *p* < 0.01, *** *p* < 0.001, and **** *p* < 0.0001.

Subsequently, we delved into the association between the expression of the POLD4 gene and signals pertaining to immunity, DNA repair, and epithelial–mesenchymal transformation. Immune-relevant signatures, mismatch-relevant signatures, and stromal-relevant signatures that have been reported were used for relevant analyses [14]. The heatmap displays the correlation between the POLD4 gene and pathway scores in pan-cancer (Figure 5A). Furthermore, we conducted a comparative analysis between the high and low POLD4 expression groups in GBM and LGG, focusing on elucidating the divergences in pathway scores. We found that in GBM and LGG, the high POLD4 group and low POLD4 group showed consistent differences in some signatures, such as CD8 T effector, immune checkpoint, antigen processing machinery, and EMT (Figure 5B,C).

### 2.5. Effect of POLD4 on Tumor Immune Microenvironment

**Figure 6:**
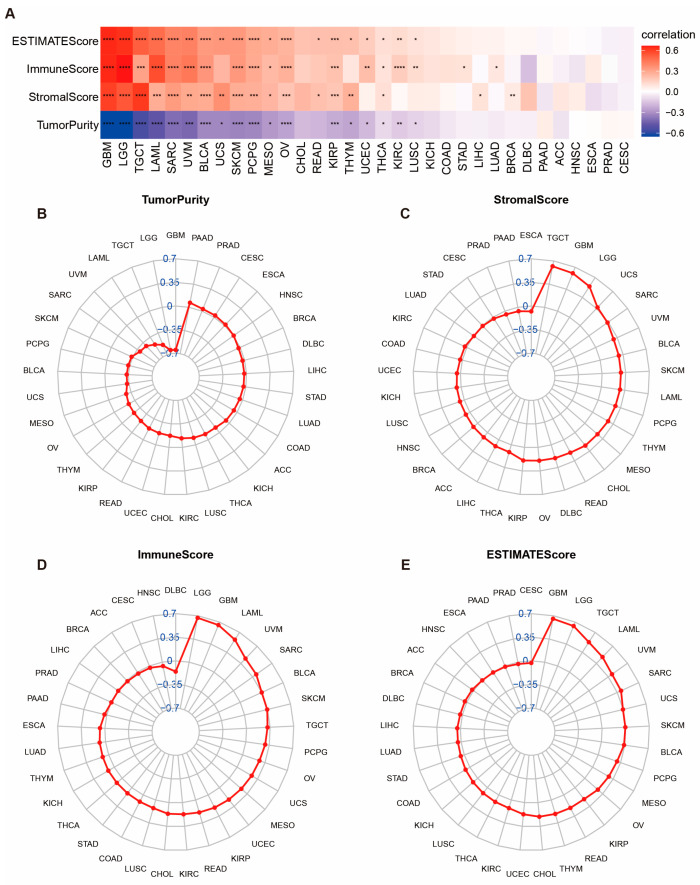
Correlation between POLD4 expression and tumor microenvironment (TME) characteristics. (**A**) Heatmap depicting the correlation between POLD4 expression and TME characteristics in pan-cancer. (**B**) Radar plot depicting the correlation between POLD4 expression and tumor purity in pan-cancer. (**C**) Radar plot depicting the correlation between POLD4 expression and stromal score in pan-cancer. (**D**) Radar plot depicting the correlation between POLD4 expression and immune score in pan-cancer. (**E**) Radar plot depicting the correlation between POLD4 expression and ESTIMATE score in pan-cancer. * *p* < 0.05, ** *p* < 0.01, *** *p* < 0.001, and **** *p* < 0.0001.

The tumor microenvironment (TME) is a sophisticated network comprising endothelial cells, fibroblasts, stromal cells, as well as innate and adaptive immune cells. By gaining insights into the intricacies of TME, we can potentially enhance therapeutic outcomes. We conducted an analysis across various cancer types to evaluate the relationship between POLD4 expression and the characteristics of the tumor microenvironment (TME). This assessment included an examination of ESTIMATE scores, tumor purity, stromal scores, and immune scores. In our investigation of the correlation between POLD4 expression and TME scores across 33 cancer types, intriguing findings emerged. It was revealed that the POLD4 expression displayed a consistent positive association with the immune score, ESTIMATE score, and stromal score across a majority of the cancer types analyzed. However, a notable inverse relationship was observed between the POLD4 expression and tumor purity (Figure 6A). To visually depict these correlations, radar plots (Figure 6B–E) was employed to illustrate the relationship between POLD4 and the ESTIMATE score, immune score, stromal score, and tumor purity. Additionally, linear correlation analysis was separately conducted for GBM (Figure S9A) and LGG (Figure S9B) to demonstrate the correlation of POLD4 with the scores.

**Figure 7:**
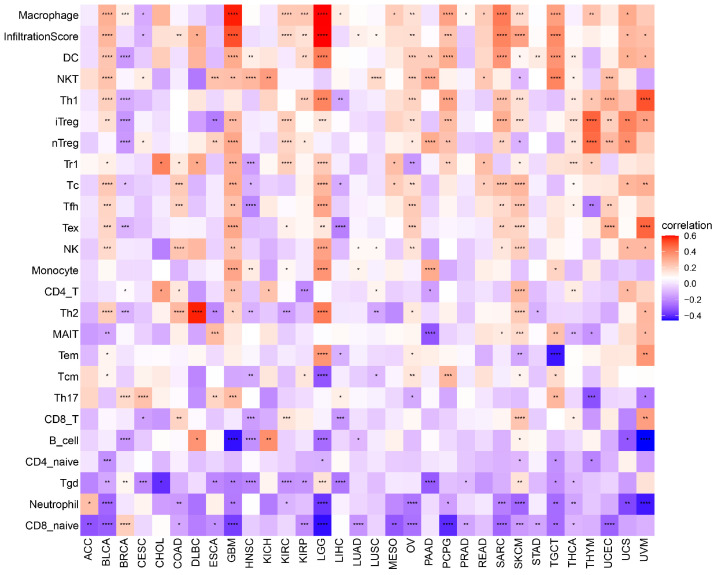
Correlation between POLD4 expression and immune cell infiltration scores collected from the ImmuCellAI database. * *p* < 0.05, ** *p* < 0.01, *** *p* < 0.001, and **** *p* < 0.0001.

Tumor-infiltrating immune cells often suffer from dysfunction, which hampers their ability to effectively control tumor growth and sometimes even aids its advancement, leading to immune evasion. To better understand the connections between POLD4 and cancer immunity, we analyzed the correlations between POLD4 expression and immune cell infiltrations. By examining the ImmuCellAI database, we found that POLD4 expression generally correlates with tumor-associated macrophages (TAMs), which play a significant role in promoting tumor growth (Figure 7). Next, we utilized the pan-cancer immune cell infiltration data from the TIMER2 database to conduct Spearman correlation analyses. The results depicted in Figure S10 revealed several significant associations. Specifically, POLD4 exhibited a positive correlation with the infiltration levels of tumor-associated fibroblasts, macrophages, monocytes, and neutrophils across various TCGA cancers. Conversely, POLD4 displayed a negative correlation with the infiltration levels of CD8^+^ T cells and NK cells.

### 2.6. Expression of POLD4 Is Associated with Immunotherapy Response

**Figure 8:**
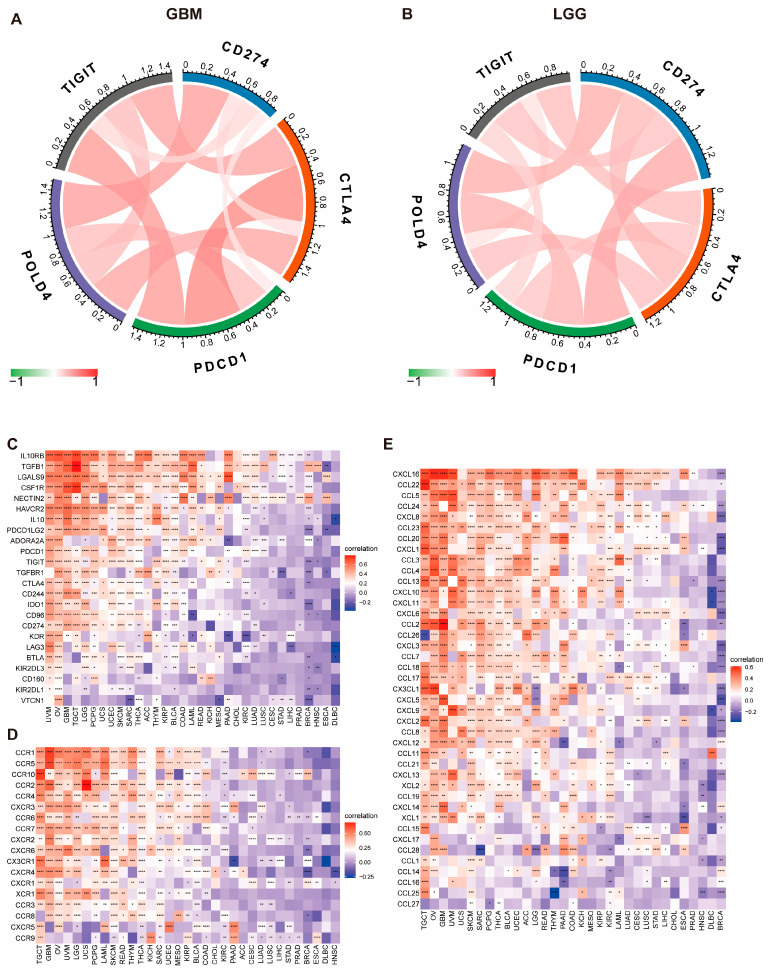
Correlation between POLD4 expression and immune-related genes. (**A**) Correlation between POLD4 expression and immune checkpoints in GBM. (**B**) Correlation between POLD4 expression and immune checkpoints in LGG. (**C**) Correlation between POLD4 expression and immunosuppressive genes in pan-cancer. (**D**) Correlation between POLD4 expression and chemokine-receptor genes in pan-cancer. (**E**) Correlation between POLD4 expression and chemokine genes in pan-cancer. * *p* < 0.05, ** *p* < 0.01, *** *p* < 0.001, and **** *p* < 0.0001.

Immunotherapies have made remarkable strides in the field of immune checkpoint therapy [15]. Therefore, we further investigated the correlation between POLD4 expression and the key factors influencing the effectiveness of immunotherapy, including immune checkpoints, immunosuppressive genes, chemokine receptors, chemokines, and MHC gene expression, spanning a comprehensive range of 33 different cancer types. Our investigation has revealed a compelling positive link between the expression of POLD4 and the presence of immune checkpoint indicators (TIGIT, CD274, CTLA4, and PDCD1) in both GBM (Figure 8A) and LGG (Figure 8B). Additionally, the results have demonstrated a favorable correlation between POLD4 expression and the majority of genes responsible for immune suppression (Figure 8C), chemokine receptors (Figure 8D), chemokines (Figure 8E), and MHC genes (Figure S11) across a diverse range of tumor types.

Immune checkpoint genes encode critical proteins like PD-1 and CTLA-4. These proteins normally serve to suppress immune responses to prevent autoimmunity or excessive immune reactions. However, certain cancer cells can evade the immune system by overexpressing these immune checkpoint proteins. Checkpoint inhibitors work by blocking this immune suppression, allowing the immune system to mount a more effective attack against cancer cells. Thus, POLD4 expression levels may influence the function of these immune checkpoint proteins, thereby impacting the treatment response. Immune-related genes encode proteins involved in regulating immune responses, such as immunosuppressive genes, chemokine receptors, and chemokines. We analyzed the correlation between POLD4 and immune-related genes across various cancer types. The elevated expression of immunosuppressive genes in association with POLD4 implies that POLD4 might contribute to an immunosuppressive microenvironment within the tumor. This could hinder the natural immune response to the tumor. The correlation with chemokine receptors and chemokines suggests that POLD4 may play a role in shaping the chemotactic signals within the tumor microenvironment. This could influence the recruitment and retention of immune cells, further affecting the immune response. The positive correlation with MHC gene expression indicates that POLD4 might be involved in modulating antigen presentation machinery. This could affect the recognition of tumor cells by the immune system.

The significance of mDNAsi score, mRNAsi score, and TMB, and their role as crucial markers in immunotherapy are widely recognized [16,17,18]. Therefore, we investigated the relationship between POLD expression and these established immunotherapy biomarkers. The results demonstrated a positive link between POLD4 expression and mDNAsi score in various cancer types, specifically OV, UVM, THYM, PRAD, and LGG. Conversely, TGCT and UCS showed a negative correlation with POLD4 expression and mDNAsi score (Figure S12A). Regarding POLD4 expression and mRNAsi score, a majority of cancer types exhibited a negative association, including GBM, TGCT, LAML, LGG, UCS, BRCA, BLCA, DLBC, READ, HNSC, LUSC, CESC, SKCM, THCA, COAD, LIHC, LUAD, and OV (Figure S12B). Notably, UCEC and KIRP showed a positive correlation between POLD4 expression and MSI, whereas UCS and STAD displayed a negative association (Figure S12C). Additionally, our analysis revealed a negative correlation between POLD4 expression and TMB in READ, STAD, and HNSC (Figure S12D).

**Figure 9:**
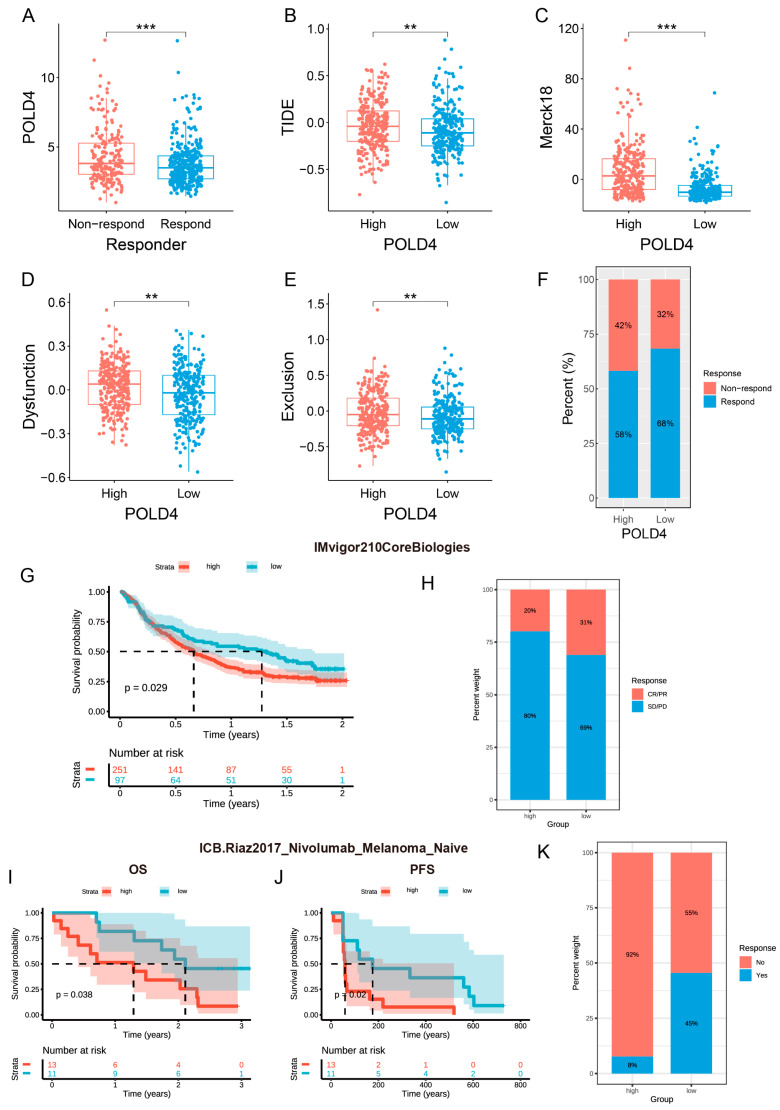
Correlation between POLD4 expression and immunotherapy response. (**A**) Comparison of POLD4 expressions between responders and non-responders analyzed using TIDE. (**B**–**E**) Comparison of TIDE score (**B**), Merck18 score (**C**), dysfunction score (**D**), and exclusion score (**E**) between POLD4-high glioma patients and POLD4-low glioma patients in the TCGA database. (**F**) Patient fraction of responders and non-responders in POLD4-high glioma patients and POLD4-low glioma patients analyzed using TIDE. (**G**) Overall survival analysis for POLD4-high patients and POLD4-low patients from the IMvigor210 clinical cohort. (**H**) Patient fraction of responders and non-responders in POLD4-high patients and POLD4-low patients from the IMvigor210 clinical cohort. (**I**,**J**) Overall survival analysis (**I**) and progression-free interval analysis (**J**) for POLD4-high patients and POLD4-low patients from the GSE91061 clinical cohort. (**K**) Patient fraction of responders and non-responders in POLD4-high patients and POLD4-low patients from the GSE91061 clinical cohort. ** *p* < 0.01 and *** *p* < 0.001.

To assess the potential of POLD4 as a prognostic indicator for patient response to immunotherapy, we first evaluated the correlation between the expression level of POLD4 in gliomas and the efficacy of immunotherapy using the TIDE algorithm. This evaluation was conducted in the TCGA, CGGA_mRNAseq_693 database, and CGGA_mRNAseq_325 database, respectively. Our results indicate that POLD4 is expressed at higher levels in non-responding patients to immunotherapy compared to responding patients (Figure 9A and Figure S13A–G). The POLD4 high-expression group has higher TIDE scores (Figure 9B and Figure S10B), Merck18 scores (Figure 9C and Figure S13C–I), dysfunction scores (Figure 9D and Figure S13D,J), and exclusion scores (Figure 9E and Figure S13E) compared to the POLD4 low-expression group. Additionally, the proportion of patients responding to immunotherapy is lower in the POLD4 high-expression group compared to the POLD4 low-expression group (Figure 9F and Figure S13F–L). Furthermore, we analyzed distinct datasets pertaining to immunotherapy in two different cancer types, namely BLCA (IMvigor210 cohort, IMvigor210CoreBiologies) and SKCM (GSE91061 cohort, ICB.Riaz2017_Nivolumab_Melanoma_Naive). The IMvigor210 cohort consisted of 348 BLCA samples, whereas the GSE91061 cohort comprised 24 melanoma samples. Noteworthy findings emerged from the anti-PD-L1 cohort (IMvigor210) group, where patients with low POLD4 expression demonstrated enhanced response to PD-L1 inhibitors characterized by prolonged OS and increased response rates (Figure 9G,H). Similarly, in the anti-PD-1 cohort (GSE91061) group, patients with low POLD4 expression displayed significantly improved OS and progression-free interval (PFI). Furthermore, patients with low POLD4 expression exhibited higher immunotherapy response rates compared to those with lower scores (Figure 9I–K).

### 2.7. Drug Sensitivity Analysis of POLD4

By utilizing data from the CTRP dataset, we examined the relationship between POLD4 expression and drug sensitivity or resistance in cancer cells. The analysis involved correlating POLD4 expression data with corresponding IC50 values of various drugs in the tumor cells. The IC50 value is an indicator of drug sensitivity, where lower values signify higher sensitivity to the drug. Employing the GSCA website (http://bioinfo.life.hust.edu.cn/GSCA/#/drug, accessed on 6 January 2023), we explored the association between the IC50 values of different compounds and POLD4 expression levels in cancer cells, summarizing the findings in Table S2. Our results indicate that the expression of POLD4 is positively correlated with the IC50 values of numerous drugs, suggesting a potential association between POLD4 expression and resistance to these drugs. In addition, we also identified several drugs whose IC50 values are negatively correlated with the expression of POLD4, such as trametinib, saracatinib, and dasatinib. These drugs could potentially be used for targeted therapy in patients with high expression of POLD4.

### 2.8. Further Analysis of POLD4’s Significance in Gliomas

**Figure 10:**
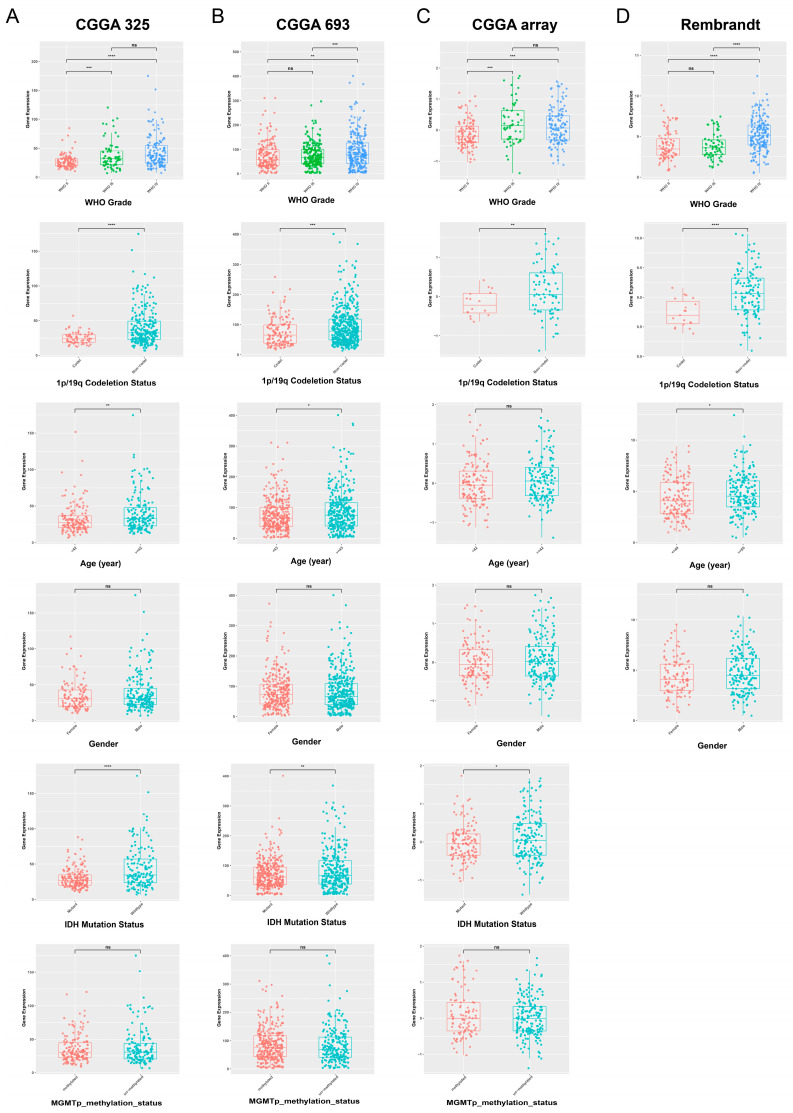
Analysis of POLD4 expression differences among subgroups with different clinical features. (**A**) POLD4 expression differences among subgroups with different clinical features analyzed in the CGGA_mRNAseq_325 database. (**B**) POLD4 expression differences among subgroups with different clinical features analyzed in the CGGA_mRNAseq_693 database. (**C**) POLD4 expression differences among subgroups with different clinical features analyzed in the CGGA_array_301 database. (**D**) POLD4 expression differences among subgroups with different clinical features analyzed in the Rembrandt database. ns. * *p* < 0.05, ** *p* < 0.01, *** *p* < 0.001, and **** *p* < 0.0001.

To further validate the significance of POLD4 in gliomas, we performed validation on other datasets of gliomas, including CGGA-mRNAseq_325, CGGA-mRNAseq_693, CGGA-mRNA_array_301, and Rembrandt cohorts. In this section, we focus on analyzing the relationship between POLD4 gene expression and clinical features of gliomas, as well as the impact of POLD4 gene expression on glioma survival. Our analysis results indicate a significant correlation between POLD4 expression and the WHO grade, 1p19q deletion status, age, and IDH mutation status of gliomas. Similar results were observed in the CGGA-mRNAseq_325 (Figure 10A), CGGA-mRNAseq_693 (Figure 10B), CGGA-mRNA_array_301 (Figure 10C), and Rembrandt cohorts (Figure 10D). Specifically, higher-grade gliomas tend to have higher POLD4 expression. WHO grade is a well-established indicator of glioma aggressiveness and patient prognosis. Our findings suggest that POLD4 could serve as a potential prognostic biomarker for glioma patients. Patients without 1p19q codeletion exhibit higher POLD4 expression compared to patients with 1p19q codeletion. Patients with 1p19q codeletion typically have better outcomes in glioma. However, our data showing higher POLD4 expression in patients without the codeletion implies that POLD4 might contribute to a more aggressive glioma phenotype in these cases. This observation could potentially inform therapeutic strategies for gliomas lacking 1p19q codeletion. Older patients show higher POLD4 expression compared to younger patients, and patients with wild-type IDH exhibit higher POLD4 expression compared to patients with IDH mutation. This suggests that POLD4 may play a role in age-related glioma development and in a subset of gliomas with IDH alterations.

**Figure 11:**
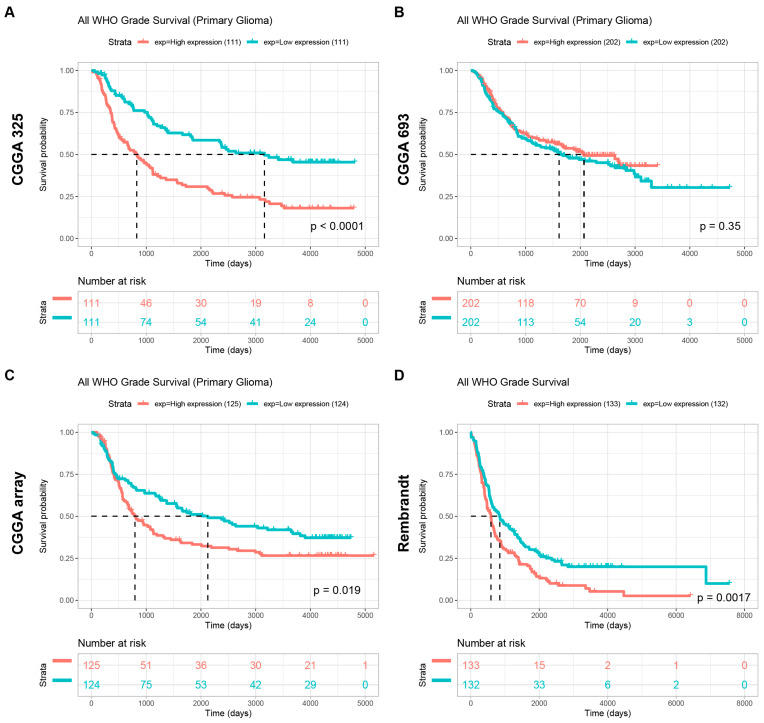
Survival analysis for POLD4-high patients and POLD4-low patients with primary glioma analyzed in CGGA_mRNAseq_325 (**A**), CGGA_mRNAseq_693 (**B**), CGGA_array_301 (**C**), and Rembrandt (**D**) database.

Furthermore, our findings elucidate a consistent trend across multiple cohorts, revealing that individuals displaying heightened POLD4 expression experience a notably shorter OS duration compared to their counterparts with lower POLD4 expression levels. The CGGA-mRNAseq_325 group yielded a *p*-value of less than 0.0001 (Figure 11A), the CGGA-mRNAseq_693 group had a *p*-value of 0.035 (Figure 11B), the CGGA-mRNA_array_301 group showed a *p*-value of 0.019 (Figure 11C), and the Rembrandt group resulted in a *p*-value of 0.0017 (Figure 11D). Taken together, these outcomes serve to reinforce the significant impact of POLD4 on the clinical characteristics and survival outcomes of glioma patients.

### 2.9. Validation of POLD4 Expression in Gliomas

**Figure 12:**
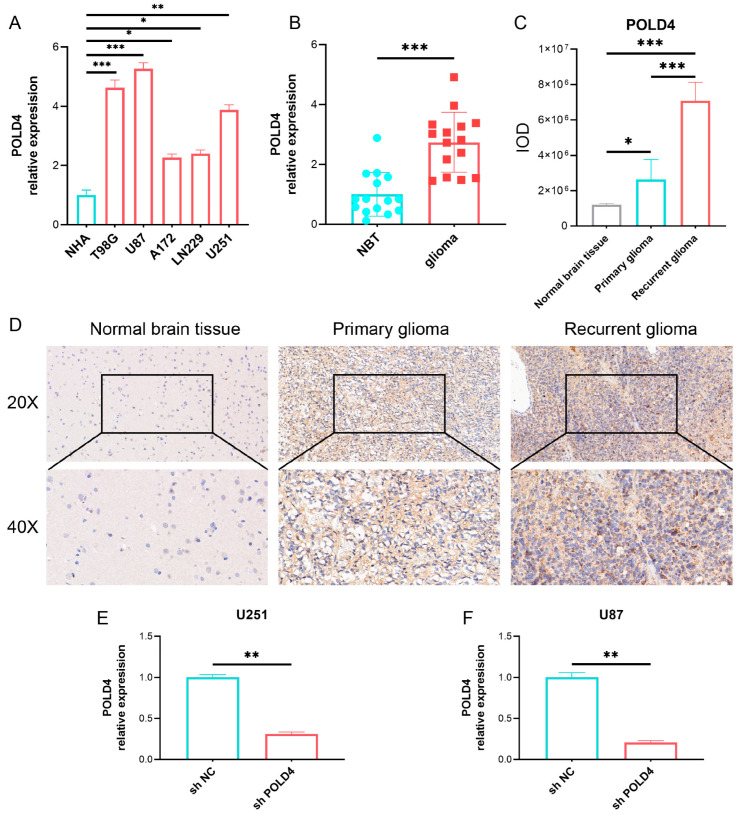
Validation of POLD4 expression in gliomas. (**A**) POLD4 expression in glioma cell lines (T98G, U87, A172, LN229, and U251) and normal human astrocyte cell lines (NHA) examined using qPCR. (**B**) POLD4 expression in glioma tissues and adjacent normal brain tissues examined using qPCR. (**C**,**D**) POLD4 expression in adjacent normal brain tissue, primary glioma, and recurrent glioma examined using immunohistochemistry. (**E**,**F**) Validating POLD4 knockdown efficiency in U251 (**E**) and U87 (**F**) cell lines using qPCR. * *p* < 0.05, ** *p* < 0.01, *** *p* < 0.001.

We conducted a thorough investigation to validate the expression of POLD4 in gliomas. Initially, we examined its presence in glioma cell lines (T98G, U87, A172, LN229, and U251) and normal human astrocyte cell lines (NHA) using qPCR. Remarkably, POLD4 showed significant overexpression in multiple glioma cell lines, particularly in U87 (Figure 12A). Subsequently, we extended our analysis to include glioma tissues and adjacent normal brain tissues. Through qPCR, we established that POLD4 is markedly upregulated in glioma tissues compared to the adjacent normal brain tissues (Figure 12B). To further verify our findings, we employed immunohistochemistry to visualize POLD4 expression in adjacent normal brain tissue, primary glioma, and recurrent glioma. Notably, glioma tissues displayed higher POLD4 expression, especially in recurrent cases (Figure 12C,D).

### 2.10. POLD4 Promotes Glioma Cell Proliferation

**Figure 13:**
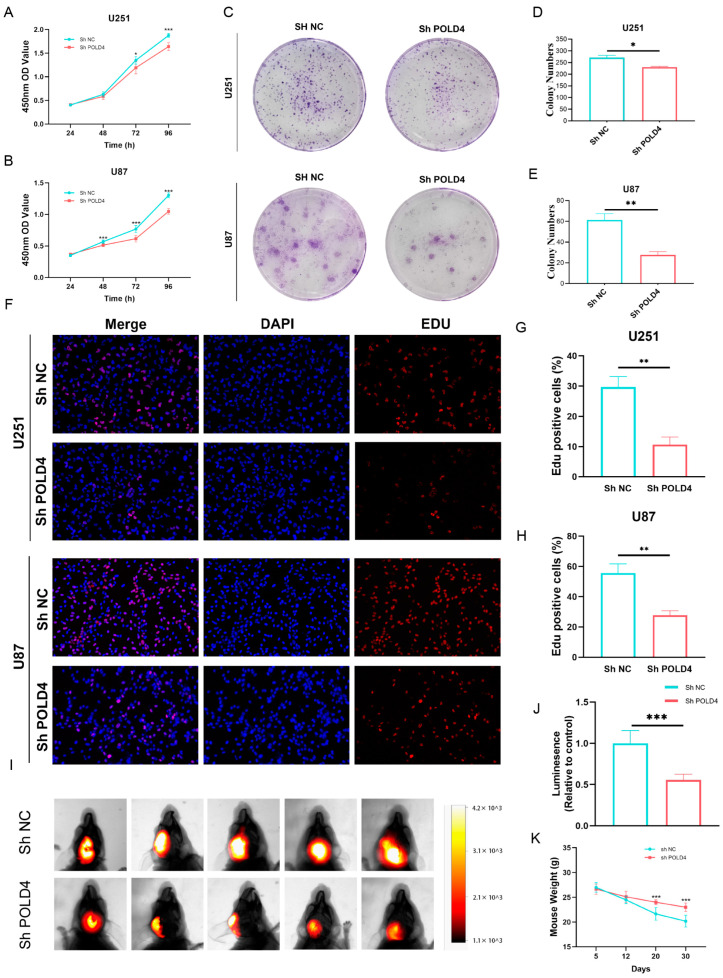
POLD4 promotes glioma cell proliferation. (**A**,**B**) CCK8 assays were employed to determine the cell viability of U251 (**A**) and U87 (**B**) cells following POLD4 knockdown. (**C**) Colony formation assays were performed to determine the clonogenic potential of U251 and U87 cells after POLD4 knockdown. (**D**,**E**) Quantitative analysis of U251 (**D**) and U87 (**E**) cell clones after POLD4 knockdown. (**F**) EDU uptake experiments were performed to determine the EDU incorporation of U251 and U87 cells after POLD4 knockdown. The microscope’s magnification is 200x. (**G**,**H**) Quantitative analysis of U251 (**G**) and U87 (**H**) EDU incorporation after POLD4 knockdown. (**I**,**J**) Bioluminescence imaging was used to monitor U87 intracranial tumorigenesis after POLD4 knockdown. (**K**) Body weights of mice in each group were recorded after U87 intracranial transplantation. * *p* < 0.05, ** *p* < 0.01, *** *p* < 0.001.

We conducted POLD4 knockdown experiments in U251 and U87 cell lines and validated the knockdown efficiency using qPCR (Figure 12E,F). CCK-8 experiments indicated that POLD4 knockdown could suppress the viability of glioma cells (Figure 13A,B). Moreover, the inhibition of POLD4 was found to impede clonogenicity, resulting in a reduced number of colonies formed, as shown by the colony formation assay (Figure 13C–E). EDU uptake experiments confirmed that POLD4 knockdown could reduce EDU incorporation in glioma cells (Figure 13F–H), indicating that POLD4 can inhibit glioma cell proliferation. In the mouse intracranial transplantation tumor experiment, we observed that the intracranial tumors in the POLD4 knockdown group were significantly smaller than those in the control group (Figure 13I,J), while the body weight was noticeably higher than that in the control group (Figure 13K), further supporting the impact of POLD4 on glioma cell proliferation in vivo.

### 2.11. Explore the Correlation of POLD4 Expression with Glioma Proliferation and Immunosuppressive Microenvironment through Histological Analysis

**Figure 14:**
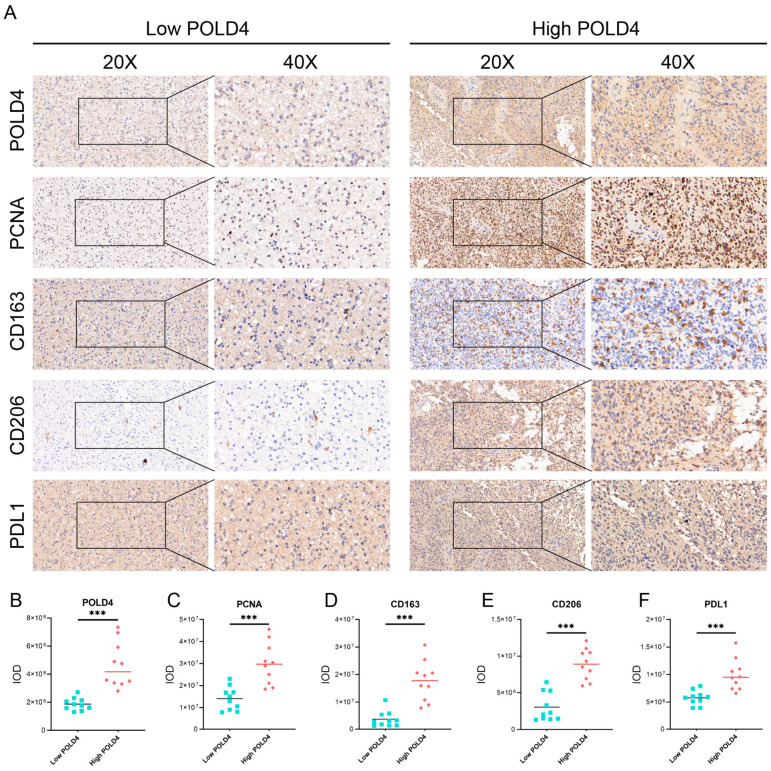
Exploring the correlation of POLD4 expression with glioma proliferation and immunosuppressive microenvironment using histological analysis. (**A**) Immunohistochemical images of the low-POLD4 group and high-POLD4 group. (**B**–**F**) The protein level of POLD4 (**B**), PCNA (**C**), CD163 (**D**), CD206 (**E**), and PDL1 (**F**) quantified using IOD in the low-POLD4 group and high-POLD4 group. *** *p* < 0.001.

As mentioned above, POLD4 influences the proliferation of glioma cells and is associated with various immune cells in gliomas, with the strongest correlation observed with macrophages. Additionally, POLD4 is correlated with the expression of immune checkpoint markers. Based on these research findings, we attempted to explore the impact of POLD4 on glioma proliferation and the immune microenvironment in glioma tissues using immunohistochemical experiments (Figure 14A). We collected tumor tissue samples from 20 glioma patients and performed immunohistochemical staining for POLD4, PCNA, CD163, CD206, and PDL1. The expression levels of each protein were quantified based on the immunohistochemistry-derived cumulative optical density (IOD) values. According to the expression levels of POLD4, we divided 20 cases of glioma into the low-POLD4 group and the high-POLD4 group (Figure 14B). PCNA, or proliferating cell nuclear antigen, is a critical protein involved in DNA replication, DNA repair, and cell cycle regulation, and is recognized as a marker of cell proliferation [19]. Moreover, PCNA has been reported as a directly interacting molecule of DNA Polymerase δ [20]. Therefore, we analyzed the expression of PCNA in the low-POLD4 group and the high-POLD4 group to assess the impact of POLD4 on glioma proliferation. Additionally, we analyzed the correlation between POLD4 and the markers of tumor-associated macrophages, CD163 and CD206, as well as the expression of the immune checkpoint PDL1 to explore the effect of POLD4 on the immunosuppressive microenvironment in gliomas. Subsequently, we compared the expression levels (IOD values) of PCNA, CD163, CD206, and PDL1 between the high POLD4 and low POLD4 groups using Student’s t-test. As expected, the high-POLD4 group showed significantly higher expression of PCNA (Figure 14C), CD163 (Figure 14D), CD206 (Figure 14E), and PDL1 (Figure 14F) compared to the low-POLD4 group, further confirming the correlation between POLD4 and glioma cell proliferation and the immunosuppressive microenvironment.

## 3. Discussion

The DNA polymerase delta (Pol δ) is a crucial player in DNA replication and genomic replication processes [21]. This enzyme has been linked to the recovery from replication fork collapse via various pathways, such as error-free template switch, break-induced replication, and error-prone translesion DNA synthesis [22]. Additionally, it has been observed that all subunits of DNA Pol δ are frequently overexpressed in human cancers [23]. However, the specific mechanism underlying DNA Pol δ amplification remains unclear, awaiting further investigation to discern its direct role in oncogenesis or its potential function as a defensive mechanism activated to bolster tolerance in the face of replication stress.

The POLD4 subunit has gained significant prominence as a pivotal factor in governing the regulation of Pol δ [24,25]. In response to DNA damage and during the transition into the S-phase, POLD4 undergoes transient degradation. This degradation leads to the transformation of Pol δ4 into a trimer, designated as Pol δ3. Cells knocked out for POLD4 exhibit a deficiency in homologous recombination (HR) repair, highlighting the requirement of Pol δ4, rather than Pol δ3, for effective HR. Furthermore, POLD4 knockout H1299 cells exhibit increased sensitivity to PARP inhibitors [26]. The inhibition of POLD4 in lung cancer cells was discovered to impede the progression of the G1-S cell cycle transition, thereby causing heightened genomic instability. Furthermore, the downregulation of POLD4 in Calu6 cells induces a blockade during the G1-S phase by suppressing the Akt-Skp2-p27 pathway [11,12]. Despite these studies reporting the role and mechanisms of POLD4 in relevant tumors, there is still vast research potential for POLD4 in cancer. Therefore, further research is needed to unveil the veil of POLD4 in tumors.

Our research findings indicate that the POLD4 gene is significantly overexpressed in 17 types of cancer compared to adjacent normal tissues. Utilizing the Univariate Cox regression analyses, we established a strong association between upregulated POLD4 expression and poorer overall survival in LGG, LAML, GBM, LUAD, PAAD, and UVM. Furthermore, a noteworthy correlation between POLD4 expression and CNA was observed. These compelling findings emphasize the significance of POLD4 as a noteworthy prognostic marker in the aforementioned tumor types.

The exact molecular mechanisms of POLD4 in cancer initiation and progression remain incompletely understood and require further research. However, the associations between POLD4 and some potential tumor-related pathways may provide valuable clues for understanding its unique functional mechanisms in cancer. Functional results from GSEA demonstrate that POLD4 has the potential to influence cancer development and progression via various mechanisms, including the regulation of immune responses such as modulating myeloid cell-mediated immunity, T-cell proliferation, leukocyte-mediated immune regulation, and involvement in immune response cell activation. Additionally, our Gene Set Variation Analysis (GSVA) reveals the extensive involvement of POLD4 in multiple pathways. Notably, POLD4 plays a role in stress-induced tumor responses, such as apoptosis pathways, P53 pathways, reactive oxygen species pathways, and hypoxia pathways, which might be correlated with the literature-reported involvement of POLD4 in DNA damage repair. These cumulative findings indicate POLD4’s multifaceted engagement in cancer development.

Our research highlights the essential role of POLD4 in cancer immunity, where the characteristics of the tumor microenvironment (TME) serve as valuable indicators for evaluating tumor cell responses to immunotherapy and influencing clinical outcomes. By utilizing ESTIMATE scores, we established positive correlations between POLD4 expression and the presence of stromal and immune cells in the TME across various types of cancer. Immunocyte populations, which play a pivotal role in the tumor immune network, significantly contribute to the development and occurrence of malignancies [27,28,29]. By investigating the ImmuCellAI and TIMER2 databases, we observed the significant impact of POLD4 on immunocyte infiltration in various cancer types, especially in LGG and GBM. Additionally, our study revealed the co-expression of POLD4 with genes encoding immunosuppressive factors, chemokine receptors, and chemokines. These comprehensive findings strongly suggest a close association between POLD4 expression and immune infiltration within tumor cells, consequently influencing patient prognosis and serving as a potential therapeutic target for the development of immunosuppressive treatments.

Cancer cells heavily depend on exhausted T cells to evade the immune system, as these T cells exhibit diminished cytokine levels and impaired effector function [30,31,32]. Numerous studies have demonstrated that targeting the underlying causes of T-cell exhaustion presents a promising therapeutic approach to enhance antitumor immunity [33,34]. Therapeutic methods involving blocking antibodies for PD-1/PD-L1 have proven effective in reactivating dormant T cells [35,36].

In our study, we discovered a positive correlation between the expression level of POLD4 and the gene markers associated with immune checkpoints. We utilized three datasets, TCGA, CGGA_mRNAseq_325, and CGGA_mRNAseq_693, and performed TIDE analysis to predict the efficacy of immune checkpoint inhibitors in glioma patients. To ensure the reliability of our conclusions, we further corroborated our findings using two clinical datasets, IMvigor210 and GSE91061. Our consistent results demonstrate that patients with low POLD4 expression exhibit a better response to immune checkpoint inhibitors. This finding suggests that high POLD4 expression may create an immune microenvironment that is less responsive to immunotherapy. Consequently, reducing the expression of POLD4 may be a viable combination strategy to enhance the effectiveness of checkpoint inhibitors by alleviating T cell exhaustion, thereby bolstering the antineoplastic response.

According to reports, POLD4 has been found to be associated with DNA damage repair [9,26], a critical process that influences the development of drug resistance [37,38]. In light of this, our investigation aimed to explore the potential correlation between POLD4 expression and drug sensitivity. The Cancer Therapeutics Response Portal (CTRP) connects the cellular characteristics of cancer cell lines with small molecule sensitivity, supporting the discovery of drugs that can be matched to patients based on predictive biomarkers. Through the Gene Set Cancer Analysis (GSCA) project, we utilized CTRP data online to perform a correlation analysis between POLD4 and drug sensitivity. Our findings indicate that POLD4 is associated with sensitivity to various drugs, particularly positively correlated with the IC50 values of multiple drugs. This intriguing finding suggests that POLD4 may play a pivotal role in mediating resistance to these drugs. Our investigation also revealed a noteworthy negative correlation between the expression of POLD4 and the IC50 values of certain drugs, including PD318088, selumetinib, pluripotin, vandetanib, IC-87114, trametinib, saracatinib, and dasatinib. Such a revelation indicates that heightened POLD4 expression might heighten cellular responsiveness to these drugs, implying a potential avenue for enhancing their therapeutic efficacy for tumor patients with elevated POLD4 levels.

To further explore the potential of POLD4 in glioma, we conducted further analysis on the expression levels of POLD4 in glioma patients with different clinical characteristics using the CGGA_mRNAseq_325, CGGA_mRNAseq_693, CGGA_array_301, and Rembrandt datasets. Additionally, we examined the differences in survival among glioma patients with high and low expression levels of POLD4. Our analysis results demonstrate a significant association between POLD4 expression levels and various malignant clinical features, as well as an impact on patient survival prognosis.

Based on the results of bioinformatic analysis, we proceeded to validate the expression of POLD4 in glioma tissues and cell lines. We found that, compared to normal astrocyte cell lines, POLD4 was expressed at higher levels in various glioma cell lines. Furthermore, compared to normal brain tissues adjacent to the tumor, POLD4 expression was significantly elevated in glioma tissues, particularly in recurrent gliomas. This substantial increase in expression suggests that POLD4 may not only be involved in glioma tumor growth but also play a role in the glioma recurrence process. By conducting knockdown experiments targeting POLD4 in glioma cell lines U251 and U87, we have unraveled compelling evidence underscoring the pivotal role of POLD4 in governing the proliferation of glioma cells. The knockdown of POLD4 exerts a profound effect on cell viability, and the clonogenic potential of glioma cells is markedly reduced upon POLD4 inhibition. Furthermore, the EDU incorporation assay found that POLD4 knockdown led to a significant reduction in EDU uptake, corroborating its role as a player in the regulation of glioma cell proliferation. POLD4 plays a crucial role in DNA replication, and we speculate that this may be a significant mechanism through which POLD4 influences the proliferation of glioma cells. Finally, we conducted a study on the correlation of POLD4 expression with cell proliferation markers PCNA, tumor-associated macrophage markers (CD163, CD206), and the immune checkpoint PDL1 in glioma tissue. Twenty glioma tissue specimens were collected, and based on POLD4 expression, they were divided into the low POLD4 expression group and the high POLD4 expression group. We found that compared to the low POLD4 expression group, the high POLD4 expression group exhibited higher levels of PCNA, CD163, CD206, and PDL1 expression in glioma tissue. This research on tissue specimens further supports the role of POLD4 in glioma cell proliferation and its impact on the glioma immune microenvironment.

**Figure 15:**
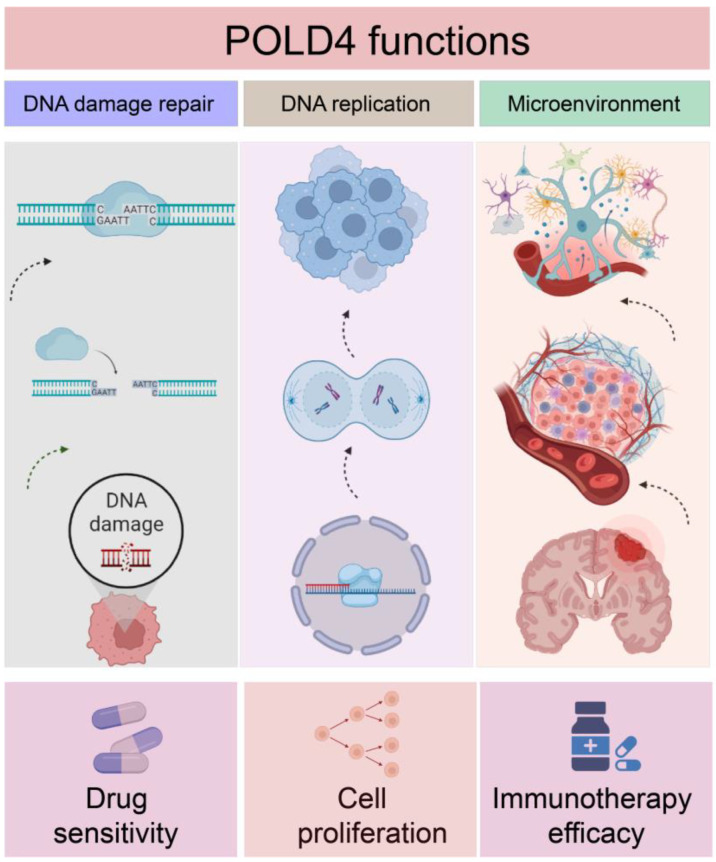
The schematic diagram illustrates the impact of POLD4 on tumor drug sensitivity, cell proliferation, and immune microenvironment.

In this study, an extensive investigation was conducted to examine the differences in POLD4 expression, prognostic significance, and its underlying functions across 33 different types of human cancers. The results demonstrated a robust link between increased POLD4 expression and adverse clinical outcomes in a diverse range of cancer types. Moreover, the study unearthed noteworthy associations between deviant POLD4 expression and an array of factors, encompassing the tumor microenvironment, efficacy of immunotherapy, drug sensitivity, and glioma cell proliferation (Figure 15).

To summarize, the elevated expression of POLD4 serves as an indicator of malignant characteristics in tumors such as gliomas, suggesting an unfavorable prognosis and contributing to the formation of an immunosuppressive microenvironment within the tumor. Utilizing a combination of POLD4-targeted cancer immunotherapy methods may offer higher efficacy compared to relying solely on a single treatment approach.

This study has several limitations. Firstly, our analysis heavily depended on publicly available datasets, which may introduce biases and limitations. Secondly, while we conducted experimental validation, we admit that the exact molecular mechanisms underlying POLD4’s role in glioma cell proliferation and the modulation of the immune microenvironment remain incompletely understood. Lastly, the extension of our findings to other tumor types is preliminary, and further research is needed to ascertain the broader implications of POLD4 in cancer biology.

## 4. Materials and Methods

### 4.1. Data Collection

The TCGA database via UCSC Xena (accessible at https://xena.ucsc.edu/, accessed on 5 December 2022) provided us with RNA sequences and clinical data from 11069 samples representing 33 different cancer types. Rembrandt (accessible via http://gliovis.bioinfo.cnio.es/, accessed on 5 December 2022) and CGGA (accessible via http://www.cgga.org.cn/, accessed on 5 December 2022) databases are also used to obtain transcriptomic and clinical information of glioma patients. We obtained gene profile data of normal human tissues from GTEx, which can be accessed through https://commonfund.nih.gov/GTEx, accessed on 5 December 2022. The POLD4 mRNA expression data for numerous cancer cell lines was acquired from the Cancer Cell Line Encyclopedia (CCLE) database, available at https://portals.broadinstitute.org/ccle/data, accessed on 5 December 2022. The DNA copy number retrieved originates from the cBioPortal database, which can be accessed at https://www.cbioportal.org/, accessed on 5 December 2022.

### 4.2. POLD4 Expression Analysis

By integrating and analyzing GTEx and TCGA databases, we conducted a comparison of POLD4 expression in different tumor types and their corresponding normal tissues. In order to obtain a thorough examination of POLD4 expression patterns, data from the TCGA and GTEx databases were used to evaluate POLD4 gene expression in a diverse set of samples, encompassing 31 normal tissues and 33 tumor tissues. We employed the CCLE database to examine POLD4 expression in tumor cell lines. Moreover, we investigated its expression discrepancies between tumors and adjacent normal tissues using data only from the TCGA database.

### 4.3. Exploration into the Copy Number Alterations

The copy number alterations (CNA) data of POLD4 in patients with various types of cancer were obtained from the cBioPortal database. We analyzed the correlation between the expression and copy number of the POLD4 gene in various types of cancer and presented the results using bar plots. The plotting was performed using the ggplot2 package. Our specific focus was on examining the correlation between POLD4 gene expression and copy number in GBM and LGG cases.

### 4.4. Prognosis Analysis

The prognostic value of POLD4 in predicting survival indicators across 33 types of cancers was assessed using Univariate Cox regression analyses, and the results are displayed in a forest plot. Moreover, to assess the prognostic significance of POLD4, the survival disparity between the high- and low-expression groups was examined utilizing Kaplan–Meier analysis.

### 4.5. Function Analysis

We conducted a correlation analysis between POLD4 and all genes in GBM and LGG separately. Heatmaps were utilized to display the expression of the top 50 genes, both positively and negatively correlated. After conducting correlation analysis, we proceeded with GSEA analysis on the GO, KEGG, and Reactome pathways in GBM and LGG. In addition, a comprehensive GSVA enrichment analysis was performed across various types of cancers. The R language “GSVA” package was utilized to derive the Hallmark pathway scores for each cancer. Visual representation of the correlation between Hallmark pathway scores and POLD4 gene expression was achieved through the use of a heatmap. To present the results in a clear manner, bar charts were employed to analyze the correlation coefficient between POLD4 gene expression and each Hallmark pathway score in GBM and LGG. Moreover, we analyzed the correlation between POLD4 gene expression and immune-relevant signature, mismatch-relevant signature, and stromal-relevant signature, which were derived from a previous literature report [14]. Principal component analysis was conducted and the first principal component (PC1) was extracted as the signature score representing corresponding biological functions. We explored the impact of POLD4 on the corresponding functions by analyzing the correlation between signature scores and POLD4 expression levels.

### 4.6. Correlation between POLD4 Expression and Tumor Microenvironment

Using the R “ESTIMATE” package, we calculated the TME score for each patient across 33 cancer types, incorporating the stromal score, immune score, ESTIMATE score, and tumor purity score. To delve deeper into our analysis, we investigated the relationship between the POLD4 expression and these aforementioned scores. Later, we acquired immune cell infiltration scores for TCGA pan-cancer patients using the ImmuCellAI platform (http://bioinfo.life.hust.edu.cn/web/ImmuCellAI/, accessed on 25 December 2022). We examined the relationship between POLD4 expression and these scores, employing the R “ggplot2” package to generate a visually informative heatmap. Finally, we assessed the relationship between POLD4 gene expression and diverse immune cell infiltrations using the TIMER2 database (http://timer.comp-genomics.org/, accessed on 25 December 2022), obtaining pan-cancer immune infiltration data.

### 4.7. Correlation between POLD4 Expression and Immunotherapy Response

We initiated our investigation into the impact of POLD4 expression on immunotherapy by investigating its association with crucial factors, including immune checkpoints, immunosuppressive genes, chemokine receptors, chemokines, and MHC gene expression. These factors have well-established roles in influencing the effectiveness of immunotherapy. Afterwards, we assessed the correlation between POLD4 expression and essential indicators for immunotherapy, such as mDNAsi score, mRNAsi score, tumor mutational burden (TMB), and microsatellite instability (MSI). Additionally, we utilized the Tumor Immune Dysfunction and Exclusion (TIDE) score to evaluate the response of glioma patients to immunotherapy [39]. In addition, our investigation encompassed an in-depth analysis of a previously documented cohort of clinical studies (IMvigor210 and GSE91061) [40,41], aiming to elucidate any plausible association between the levels of POLD4 expression and the response to immunotherapy.

### 4.8. Drug Sensitivity Analysis

In order to examine the associations between POLD4 and various small-molecule drugs, we leveraged the Cancer Therapeutics Response Portal (CTRP, accessible at https://portals.broadinstitute.org/ctrp/, accessed on 6 January 2023) datasets to calculate the Pearson correlation coefficients. These coefficients were used to evaluate the relationship between POLD4 expression and the corresponding drug sensitivity.

### 4.9. Clinical Sample Collection

Clinical samples were collected from glioma patients who underwent surgical resection. Informed consent was obtained from all participants, and the study was conducted following the guidelines and approval of Union Hospital, Tongji Medical College, Huazhong University of Science and Technology.

### 4.10. Quantitative Real-Time PCR (qPCR)

RNAiso Plus (Takara, 9109) was used to isolate total RNA from the experimental samples, following the manufacturer’s instructions. Subsequently, the extracted RNA was employed to synthesize complementary DNA (cDNA) via a reverse transcription kit (Vazyme, R323). For qPCR, a SYBR Green-based master mix (Vazyme, Q311-02/03) was utilized. The 2^−ΔΔCT^ method was applied to quantify the gene expression levels, with GAPDH serving as the internal control for data normalization. Primers for POLD4 (forward, TGTGAAGAGGAGGGAGGGG, reverse, TGCCAGGCCAGGTCAAACT) and GAPDH (forward, AAAAGCATCACCCGGAGGAGAA, reverse, AAGGAAATGAATGGGCAGCCG) were used.

### 4.11. Immunohistochemistry

After deparaffinization and rehydration of tissue sections, antigen retrieval was performed. To quench endogenous peroxidase activity, a solution of methanol containing 3% hydrogen peroxide was utilized, while 3% bovine serum albumin was employed to block non-specific binding. Primary antibodies specific to the target proteins were applied to the sections, and after incubation, a secondary antibody (HRP polymer) was used for detection. The stained tissue sections were examined under a microscope.

### 4.12. Cell Culture and shRNA Interference

Cell lines relevant to the study were cultured in DMEM supplemented with serum and antibiotics. Incubation was carried out in a humidified incubator at 37 °C with 5% CO_2_. Lentiviral particles containing targeted ShRNA constructs for POLD4 or non-targeting control ShRNA were transduced into the cells for ShRNA interference experiments. Stable knockdown cell lines were established using puromycin selection. Knockdown efficiency was subsequently validated using qPCR.

### 4.13. Cell Proliferation Assays

CCK-8, colony formation, and EdU staining assays were performed to explore the effect of POLD4 on cell proliferation. Cell viability was assessed using CCK-8 assays (Biosharp, Hefei, China, BS350) following the manufacturer’s instructions. Cells were seeded in 96-well plates and treated with different conditions as required. The absorbance at the specified wavelength was measured using a microplate reader. In the colony formation assay, cells were plated at a low density of 1000 cells/well and allowed to grow for 2 weeks. Afterward, cells were fixed, and stained, and the number of colonies was counted. For EdU staining assays, cells were cultured with EDU, a nucleoside analog of thymidine, which is incorporated into actively replicating DNA. After 1–3 h, the cells were fixed, and the incorporated EDU was detected using fluorescence microscopy.

### 4.14. Xenograft Mouse Model

To establish an orthotopic glioma model, the researchers used six-week-old female BALB/c nude mice. In brief, we injected a total of 5 × 10^5^ U87MG cells, which stably expressed firefly luciferase (Fluc), into the mouse brain utilizing a stereotactic instrument. Subsequently, the progression of the tumor was monitored on the 30th day post-tumor implantation using bioluminescence imaging (Bruker Corporation, Billerica, MA, USA).

### 4.15. Statistical Analysis

The gene expression data underwent normalization using a log2 transformation with the formula [TPM (Transcripts per million) + 1]. To compare the two groups, the statistical analysis employed Student’s t-test. For comparisons involving more than two groups, the non-parametric Kruskal–Wallis test and the parametric one-way ANOVA test were utilized. Spearman’s test was employed to analyze the correlation between the two variables. The R software (Version 4.2.2) was used for bioinformatics analysis, while GraphPad Prism 9.0 software was utilized for analyzing the experimental data. A significance level below *p* < 0.05 was considered statistically significant.

## Data Availability

The datasets presented in this study can be found in online repositories. The locations of the websites can be found in the article.

## Supplementary Materials

The supporting information can be downloaded at: https://www.mdpi.com/article/10.3390/ijms241813919/s1.
