# Supplementary material for: POLD4 Promotes Glioma Cell Proliferation and Suppressive Immune Microenvironment: A Pan-Cancer Analysis Integrated with Experimental Validation
Source: Int J Mol Sci. 2023 Sep 10;24(18):13919. doi: 10.3390/ijms241813919 (PMC10530695; doi:10.3390/ijms241813919)
Supplement: Supplementary file 1 [file ijms-24-13919-s001.zip › Supplementary Figure S1-13.pdf]

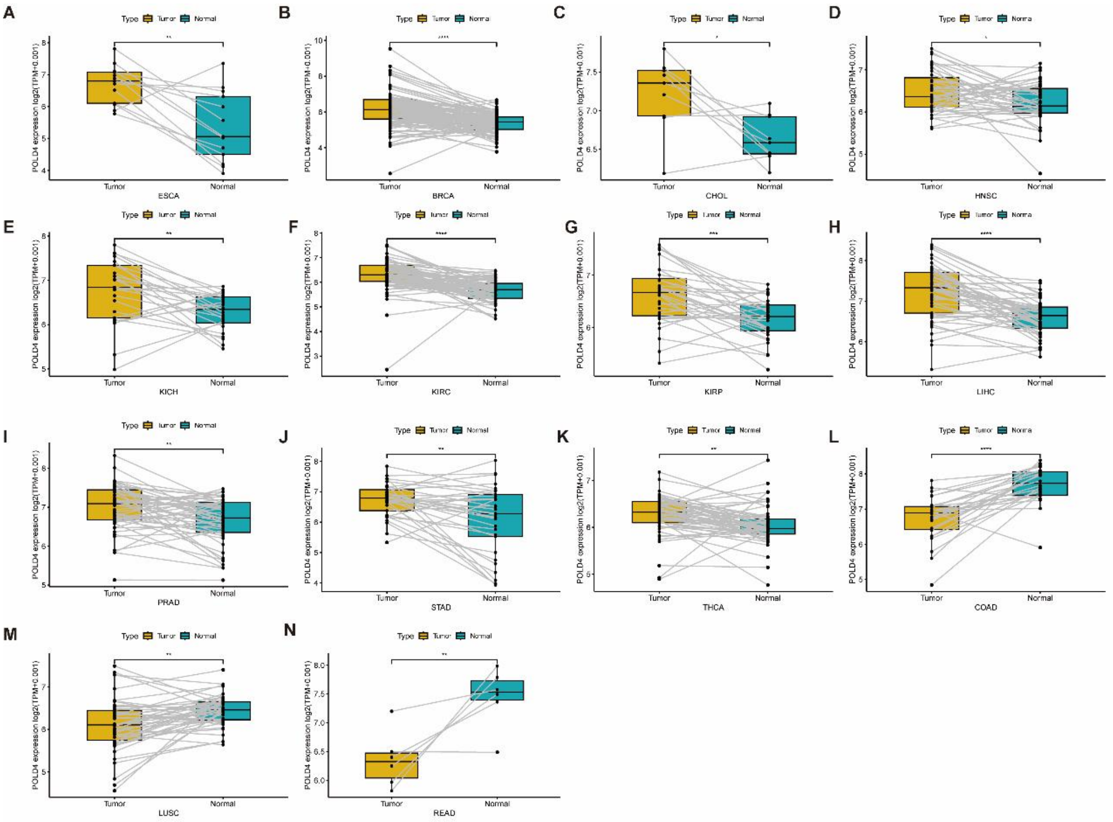

Figure S1. POLD4 expression levels in paired tumor and adjacent normal tissues. (A-K) Differential expression of POLD4 in paired tumor and adjacent normal tissues, with elevated expression of POLD4 observed in tumor tissues. (L-N) Differential expression of POLD4 in paired tumor and adjacent tissues, with low expression of POLD4 observed in tumor tissues. ns p>0.05, *p<0.05, **p<0.01, ***p<0.001, ****p<0.0001.

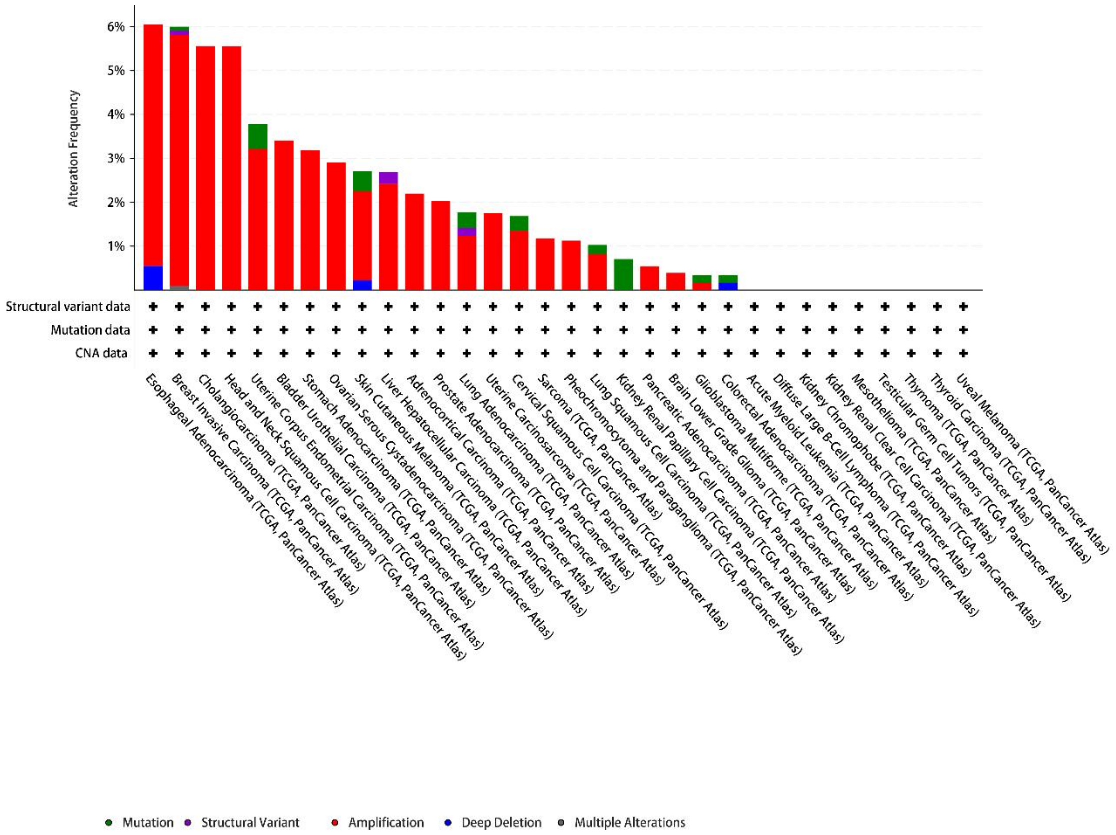

Figure S2: POLD4 mutation and copy number alteration (CNA) status assessed across various cancer types using the cBioportal database.

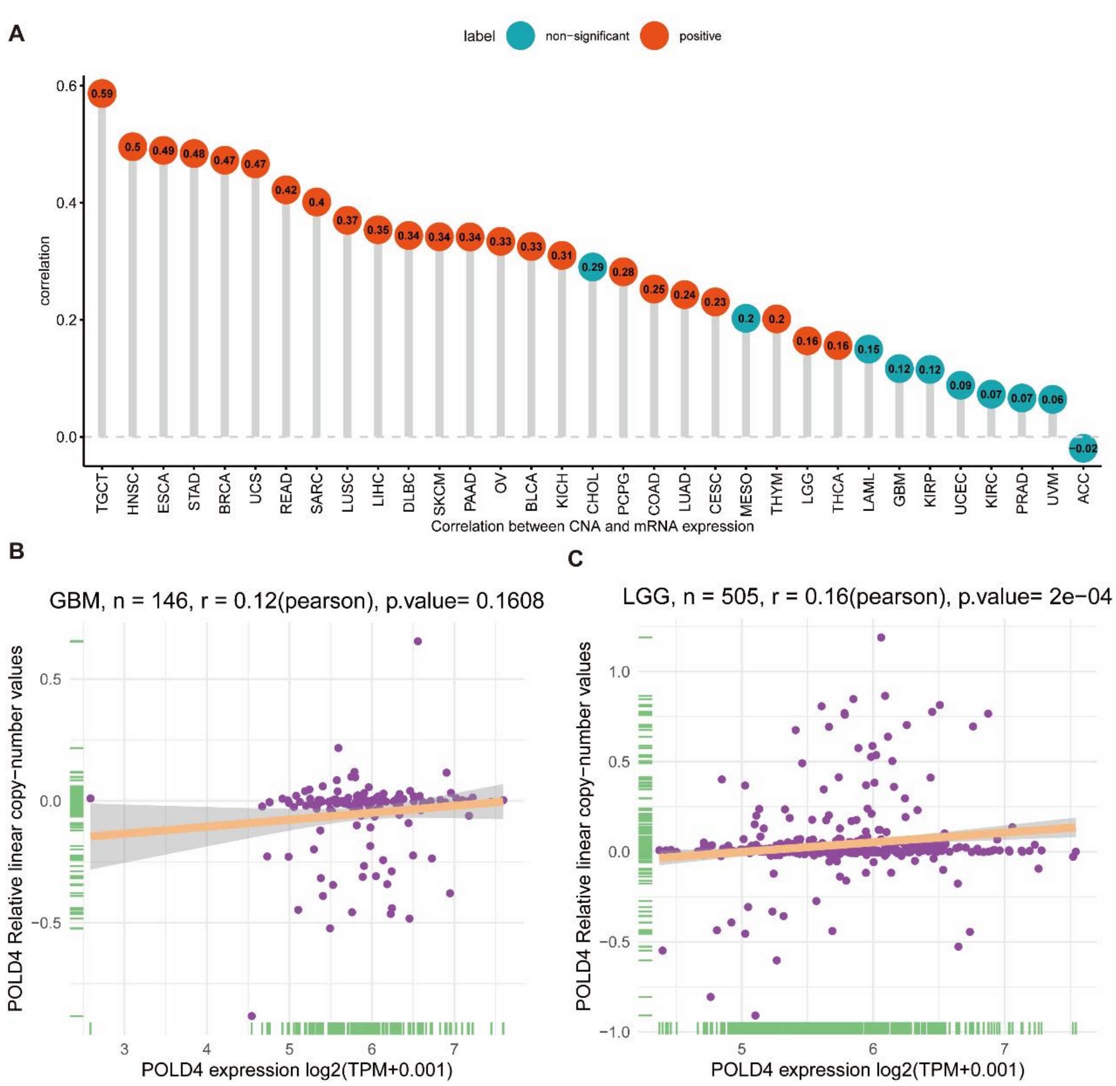

Figure S3. Correlation between POLD4 mRNA expression and copy number alterations. (A) Correlation between POLD4 mRNA expression and copy number alterations in pan-cancer. Green indicates no statistical difference, while red indicates a positive correlation. (B) Correlation between POLD4 mRNA expression and copy number alterations in GBM. (C) Correlation between POLD4 mRNA expression and copy number alterations in LGG.

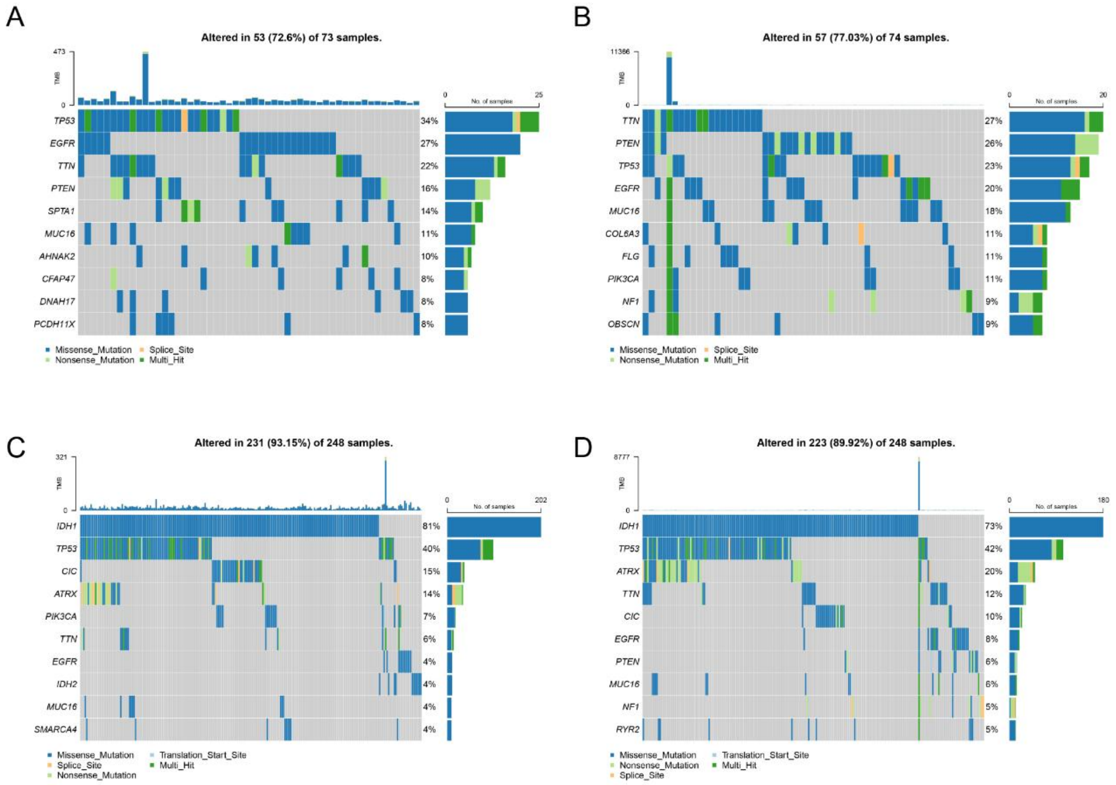

Figure S4: Gene mutation differences in the POLD4-low group and POLD4-high group. (A) Top 10 mutated genes in POLD4-low GBM group. (B) Top 10 mutated genes in POLD4-high GBM group. (C) Top 10 mutated genes in POLD4-low LGG group. (D) Top 10 mutated genes in POLD4-high LGG group.

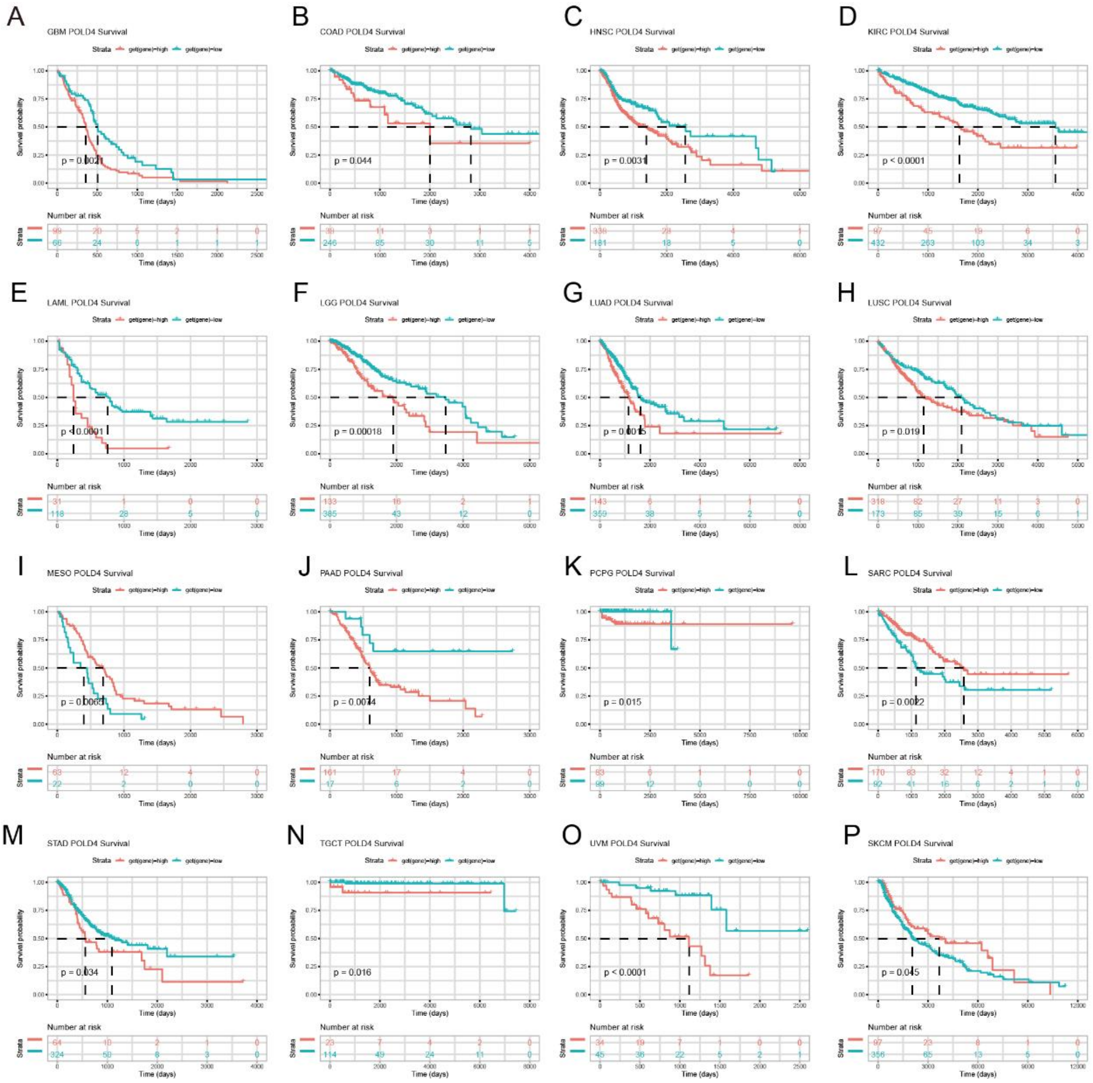

Figure S5. Kaplan–Meier analysis of the association between POLD4 expression and overall survival in GBM (A), COAD (B), HNSC (C), KIRC (D), LAML (E), LGG (F), LUAD (G), LUSC (H), MESO (I), PAAD (J), PCPG (K), SARC (L), STAD (M), TGCT (N), UVM (O), and SKCM (P).

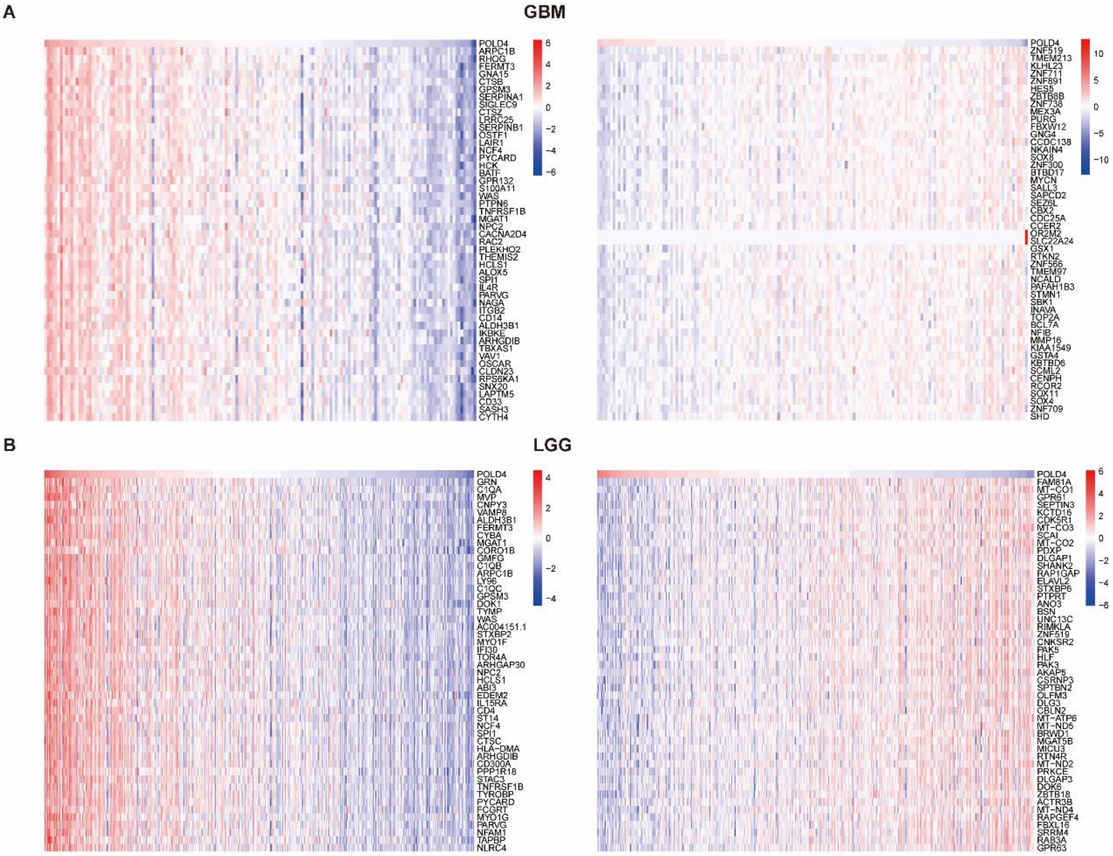

Figure S6. Correlation analysis of POLD4 with other genes. (A) Top 50 genes positively associated POLD4 in GBM. (B) Top 50 genes negatively associated POLD4 in GBM. (C) Top 50 genes positively associated POLD4 in LGG. (D) Top 50 genes negatively associated POLD4 in LGG.

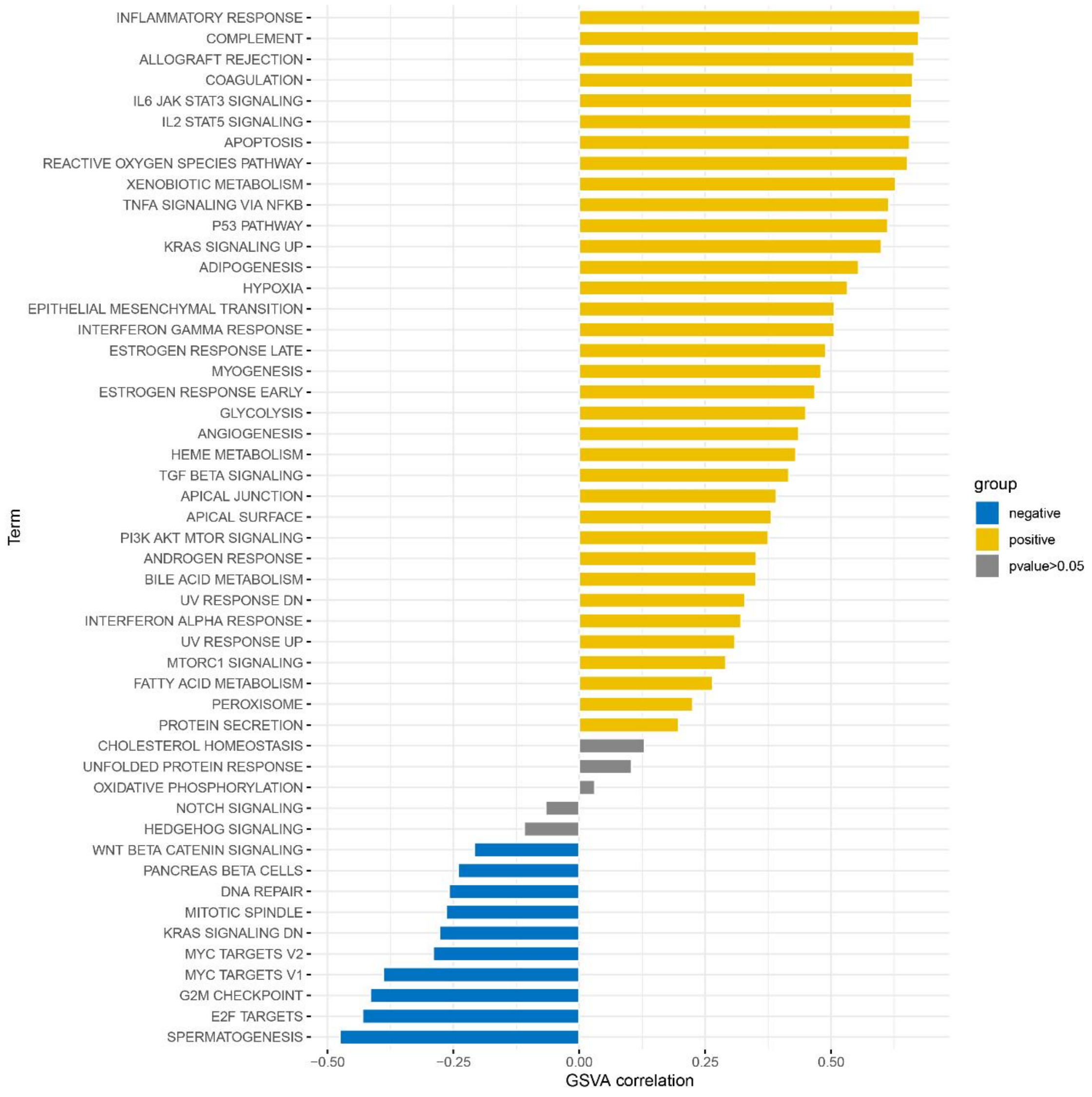

Figure S7. GSVA analysis for POLD4 in GBM.

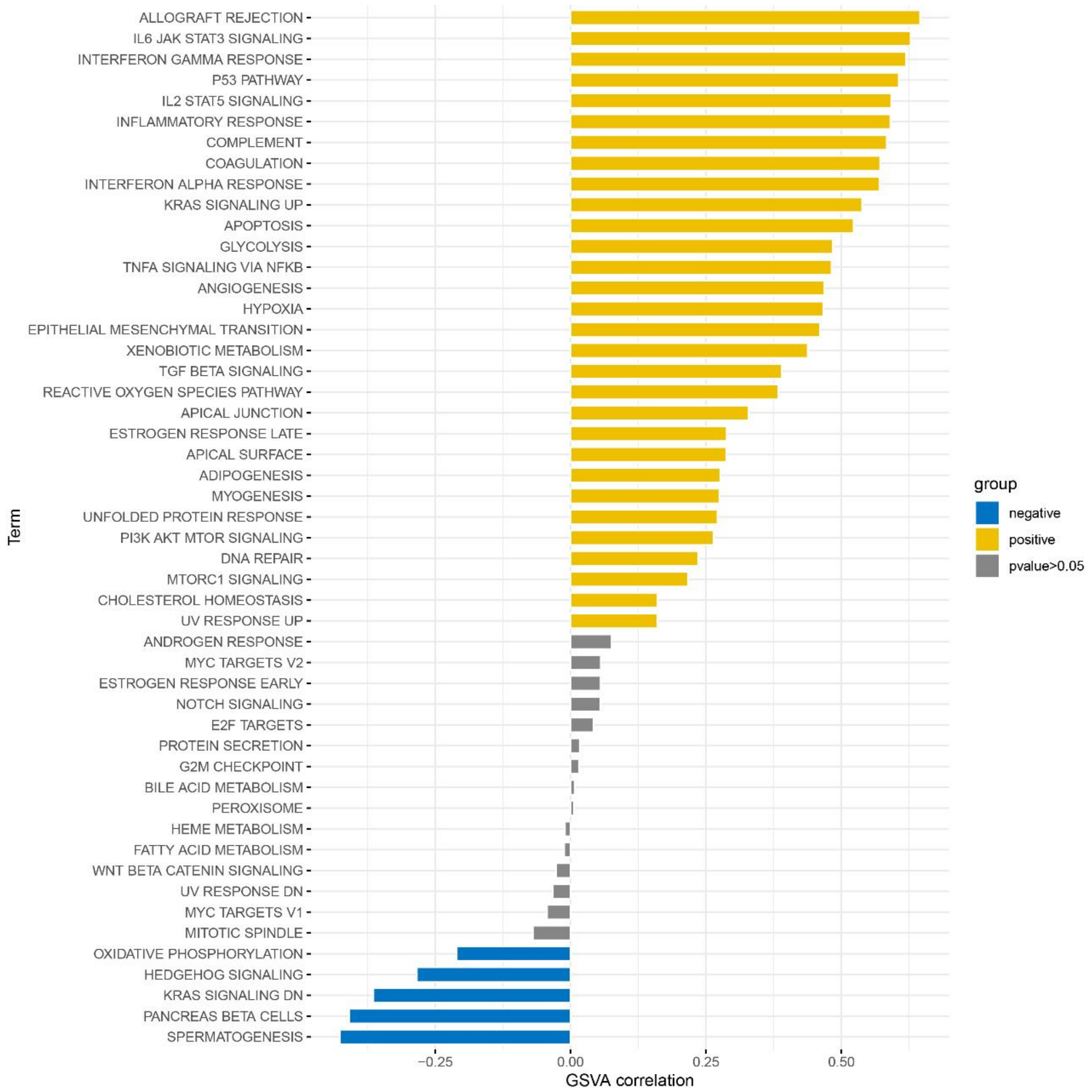

Figure S8. GSVA analysis for POLD4 in LGG.

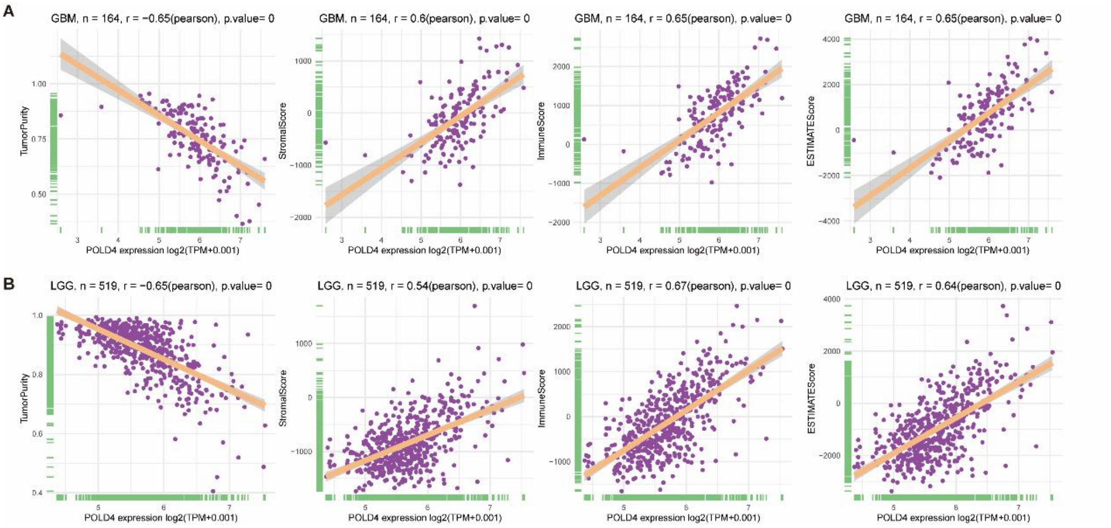

Figure S9: Correlation analysis between POLD4 expression and TME characteristics in GBM (A) and LGG (B).

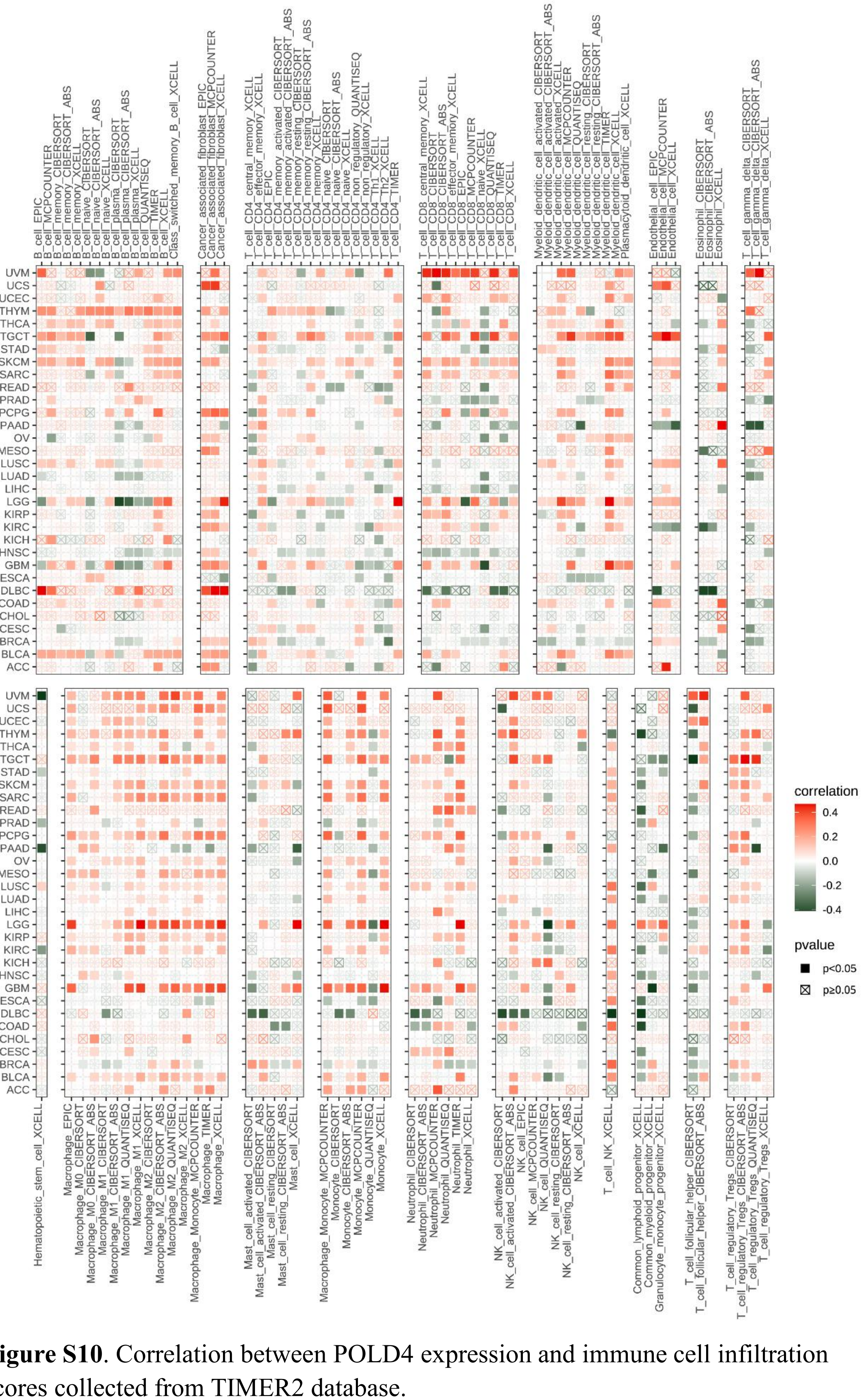

Figure S10. Correlation between POLD4 expression and immune cell infiltration scores collected from TIMER2 database.

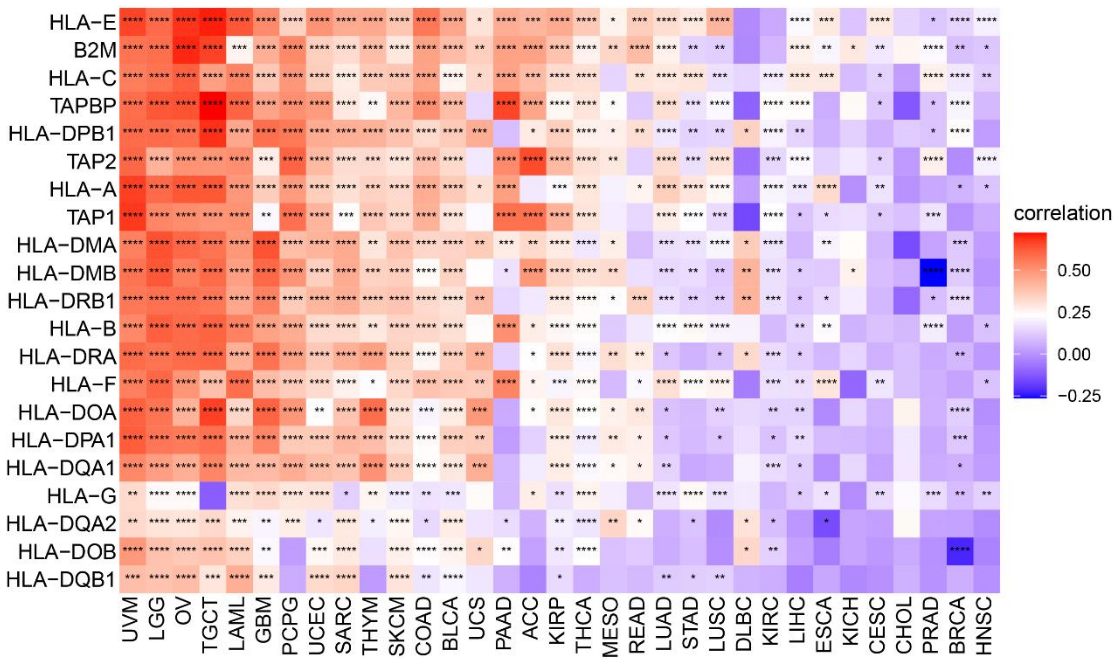

Figure S11: Correlation analysis between POLD4 expression and MHC genes in pan-cancer.

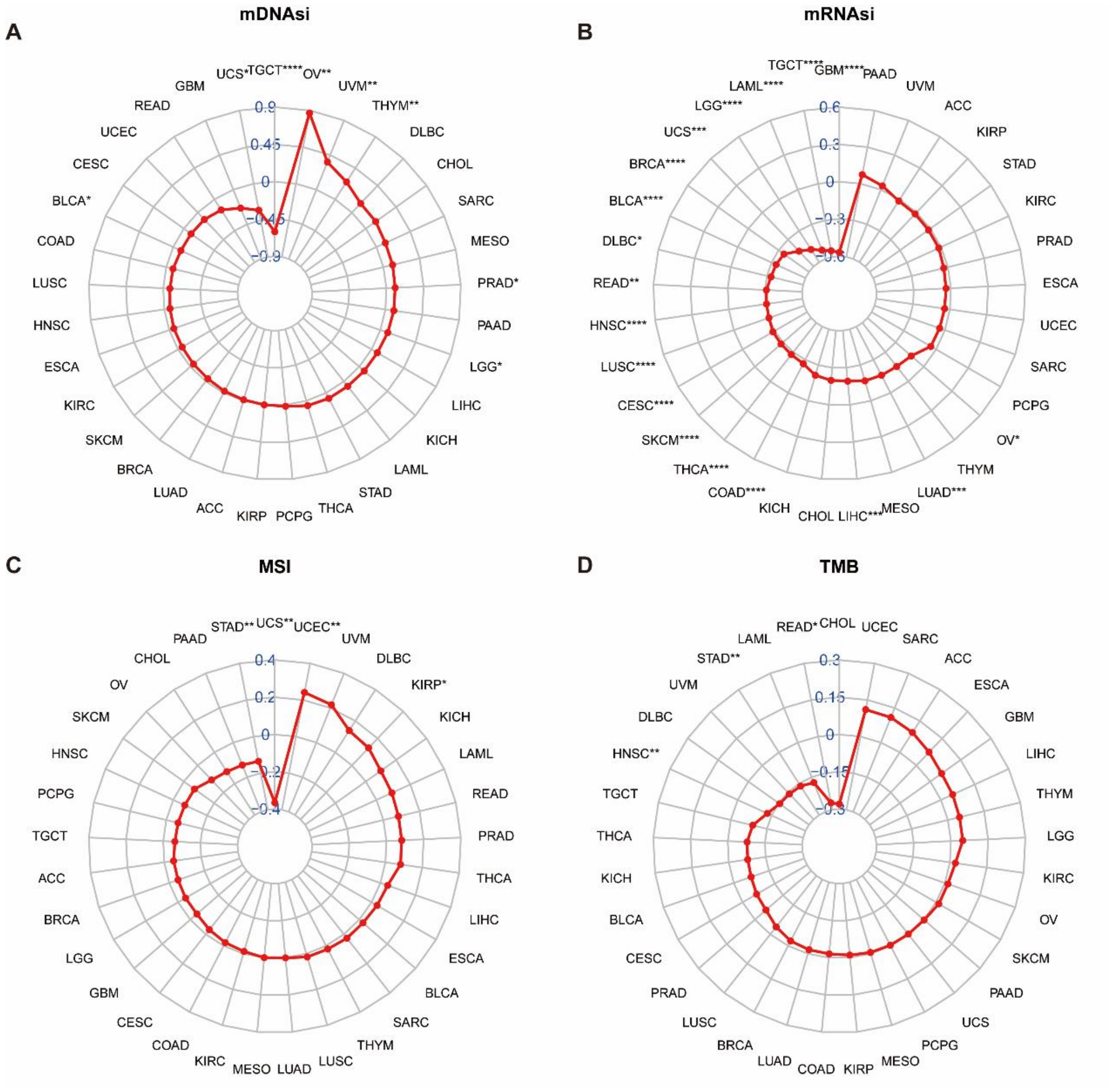

Figure S12: Correlation between POLD4 expression and mDNAsi score, mRNAsi score Microsatellite Instability (MSI), tumor mutational burden (TMB). (A-D) Radar plot depicting the correlation between POLD4 expression and mDNAsi score (A), mRNAsi score (B), MSI (C), TMB (D).

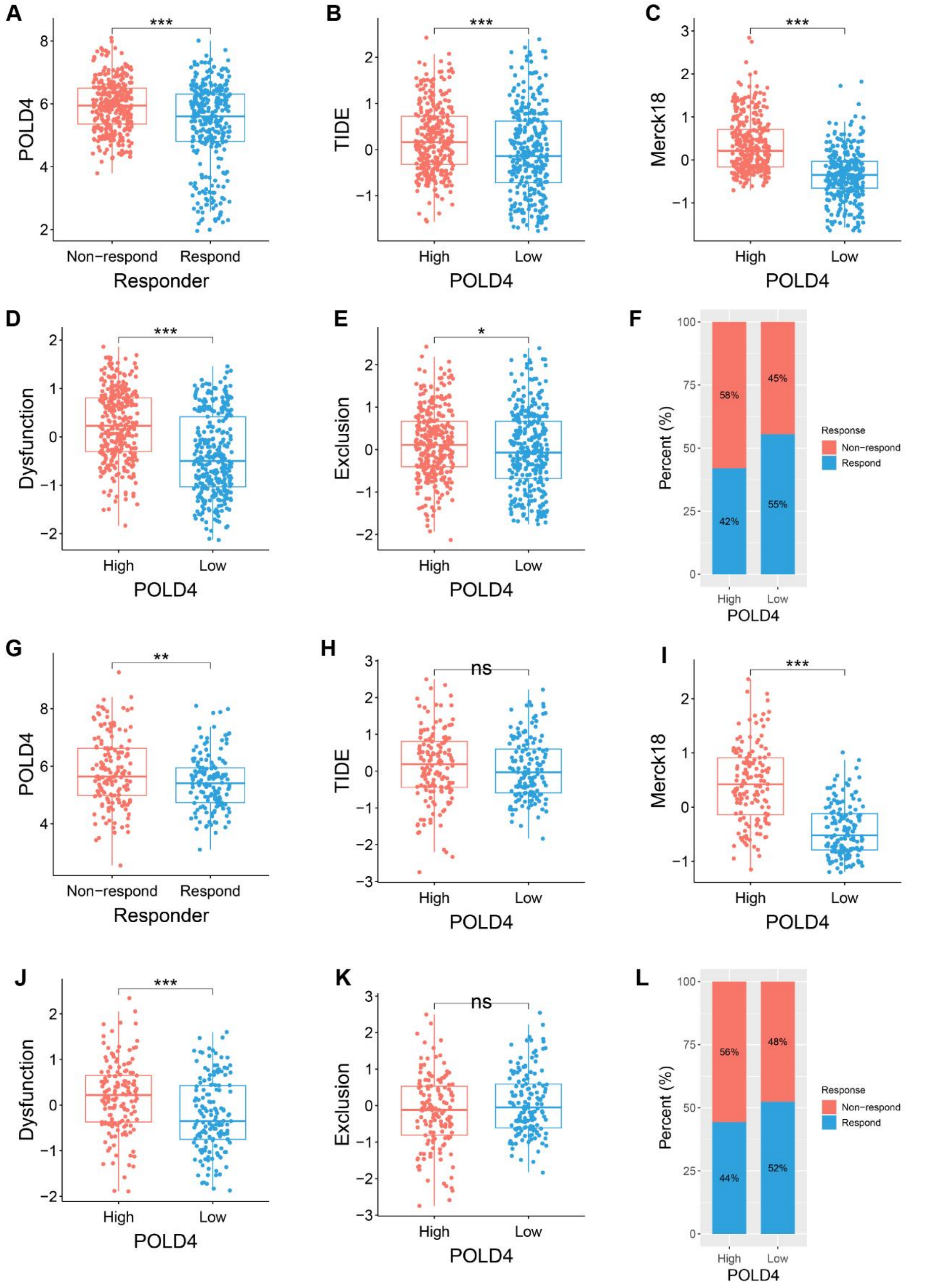

Figure S13: Correlation between POLD4 expression and immunotherapy response analyzed with TIDE. (A) Comparison of POLD4 expression between responders and non-responders in CGGA_mRNAseq_693 database. (B-E) Comparison of TIDE score (B), Merck18 score (C), dysfunction score (D), and exclusion score (E) between POLD4-high glioma patients and POLD4-low glioma patients in CGGA_mRNAseq_693 database. (F) Patient fraction of responders and

non-responders in POLD4-high glioma patients and POLD4-low glioma patients in CGGA_mRNAseq_693 database. (G) Comparison of POLD4 expression between responders and non-responders in CGGA_mRNAseq_325 database. (H-K) Comparison of TIDE score (H), Merck18 score (I), dysfunction score (J), and exclusion score (K) between POLD4-high glioma patients and POLD4-low glioma patients in CGGA_mRNAseq_325 database. (L) Patient fraction of responders and non-responders in POLD4-high glioma patients and POLD4-low glioma patients in CGGA_mRNAseq_325 database.
