# Supplementary material for: POLD4 Promotes Glioma Cell Proliferation and Suppressive Immune Microenvironment: A Pan-Cancer Analysis Integrated with Experimental Validation
Source: Int J Mol Sci. 2023 Sep 10;24(18):13919. doi: 10.3390/ijms241813919 (PMC10530695; doi:10.3390/ijms241813919)
Supplement: Supplementary file 1 [file ijms-24-13919-s001.zip › Table S2.pdf]

| ID | drug | cor | fdr | Label |
|---|---|---|---|---|
| POLD4 | GSK-J4 | 0.508649245 | 0.00021928 | positive |
| POLD4 | SB-225002 | 0.348892041 | 8.97871E-23 | positive |
| POLD4 | BRD-K01737880 | 0.346988361 | 0.042775089 | positive |
| POLD4 | LY-2183240 | 0.333031045 | 2.29621E-20 | positive |
| POLD4 | FQI-1 | 0.332094786 | 1.42948E-10 | positive |
| POLD4 | ceranib-2 | 0.330959716 | 2.43856E-20 | positive |
| POLD4 | ELCPK | 0.323003709 | 5.26441E-09 | positive |
| POLD4 | isoevodiamine | 0.316311452 | 2.82705E-18 | positive |
| POLD4 | vincristine | 0.312930364 | 8.2519E-19 | positive |
| POLD4 | YK 4-279 | 0.312625241 | 5.3884E-18 | positive |
| POLD4 | KX2-391 | 0.299401553 | 3.23234E-16 | positive |
| POLD4 | triazolothiadiazine | 0.298976611 | 7.08063E-17 | positive |
| POLD4 | BI-2536 | 0.297925197 | 8.03554E-17 | positive |
| POLD4 | Compound 7d-cis | 0.296483438 | 2.4172E-13 | positive |
| POLD4 | axitinib | 0.293507455 | 1.31325E-15 | positive |
| POLD4 | CD-437 | 0.293287236 | 1.19147E-15 | positive |
| POLD4 | omacetaxine mepesuccinate | 0.291141259 | 2.24547E-11 | positive |
| POLD4 | ML311 | 0.291054311 | 7.28681E-16 | positive |
| POLD4 | CHM-1 | 0.290083824 | 1.61106E-15 | positive |
| POLD4 | SCH-79797 | 0.285346957 | 1.13044E-14 | positive |
| POLD4 | FQI-2 | 0.28468719 | 3.72911E-15 | positive |
| POLD4 | parbendazole | 0.284321018 | 2.57048E-15 | positive |
| POLD4 | BRD-K70511574 | 0.282552501 | 1.34136E-14 | positive |
| POLD4 | SB-743921 | 0.281799327 | 4.41794E-15 | positive |
| POLD4 | neopeltolide | 0.281753805 | 0.000926286 | positive |
| POLD4 | GSK461364 | 0.278934667 | 3.23776E-14 | positive |
| POLD4 | MK-1775 | 0.278572682 | 7.45683E-14 | positive |
| POLD4 | tivantinib | 0.277792905 | 2.85871E-07 | positive |
| POLD4 | BRD-A94377914 | 0.277426895 | 1.31953E-07 | positive |
| POLD4 | PL-DI | 0.277260204 | 6.2788E-14 | positive |
| POLD4 | teniposide | 0.275703889 | 9.12838E-08 | positive |
| POLD4 | mitomycin | 0.27354978 | 1.15978E-13 | positive |
| POLD4 | BRD-K34222889 | 0.269612146 | 1.95247E-13 | positive |
| POLD4 | cerulenin | 0.269524402 | 3.85611E-13 | positive |
| POLD4 | nakiterpiosin | 0.266050764 | 5.4434E-13 | positive |
| POLD4 | BRD-K66453893 | 0.265952413 | 4.96696E-13 | positive |
| POLD4 | belinostat | 0.26513859 | 7.20738E-07 | positive |
| POLD4 | rigosertib | 0.26511902 | 1.49661E-12 | positive |
| POLD4 | WP1130 | 0.264347394 | 4.58843E-12 | positive |
| POLD4 | vorinostat | 0.263035026 | 1.09018E-12 | positive |
| POLD4 | panobinostat | 0.262941517 | 4.97094E-13 | positive |
| POLD4 | doxorubicin | 0.262369404 | 3.46236E-13 | positive |
| POLD4 | indisulam | 0.26215996 | 4.74246E-12 | positive |
| POLD4 | entinostat | 0.257475917 | 4.48536E-12 | positive |
| POLD4 | PX-12 | 0.25740652 | 3.4685E-12 | positive |

| | | | | |
|---|---|---|---|---|
| POLD4 | necrosulfonamide | 0.256808928 | 1.37236E-08 | positive |
| POLD4 | KPT185 | 0.253201684 | 4.86789E-09 | positive |
| POLD4 | MST-312 | 0.24964306 | 2.5878E-11 | positive |
| POLD4 | NSC95397 | 0.249045538 | 2.57162E-10 | positive |
| POLD4 | BRD-K26531177 | 0.246885349 | 2.49866E-10 | positive |
| POLD4 | piperlongumine | 0.245887804 | 2.04673E-11 | positive |
| POLD4 | dinaciclib | 0.245568628 | 6.82501E-06 | positive |
| POLD4 | NVP-231 | 0.244910301 | 4.47063E-11 | positive |
| POLD4 | etoposide | 0.23976899 | 6.90159E-11 | positive |
| POLD4 | daporinad | 0.238783626 | 7.36944E-09 | positive |
| POLD4 | ISOX | 0.238452543 | 1.26528E-10 | positive |
| POLD4 | curcumin | 0.237945889 | 1.72436E-10 | positive |
| POLD4 | tipifarnib-P2 | 0.23789817 | 5.21814E-06 | positive |
| POLD4 | BRD-K97651142 | 0.23721542 | 2.09799E-09 | positive |
| POLD4 | phloretin | 0.235337982 | 4.65263E-10 | positive |
| POLD4 | ciclopirox | 0.235134203 | 1.24432E-10 | positive |
| POLD4 | barasertib | 0.234438132 | 3.99165E-10 | positive |
| POLD4 | cucurbitacin I | 0.234344929 | 1.11148E-09 | positive |
| POLD4 | tacedinaline | 0.234254639 | 1.49288E-07 | positive |
| POLD4 | narciclasine | 0.234007768 | 4.07696E-10 | positive |
| POLD4 | NSC632839 | 0.23281594 | 3.51389E-10 | positive |
| POLD4 | KHS101 | 0.231469839 | 6.84022E-09 | positive |
| POLD4 | 3-Cl-AHPC | 0.231414878 | 4.83744E-10 | positive |
| POLD4 | BRD1812 | 0.229508425 | 1.91303E-09 | positive |
| POLD4 | oligomycin A | 0.22922357 | 2.31383E-09 | positive |
| POLD4 | PAC-1 | 0.229084049 | 2.23188E-09 | positive |
| POLD4 | Bax channel blocker | 0.228146109 | 2.96193E-08 | positive |
| POLD4 | manumycin A | 0.226419133 | 1.00801E-09 | positive |
| POLD4 | niclosamide | 0.226223437 | 1.26807E-08 | positive |
| POLD4 | paclitaxel | 0.226042652 | 1.47064E-09 | positive |
| POLD4 | STF-31 | 0.225205625 | 4.08874E-09 | positive |
| POLD4 | leptomycin B | 0.223958899 | 6.78583E-10 | positive |
| POLD4 | B02 | 0.223243922 | 8.31551E-09 | positive |
| POLD4 | Repligen 136 | 0.222620987 | 3.34131E-08 | positive |
| POLD4 | apicidin | 0.220391843 | 1.90076E-09 | positive |
| POLD4 | SCH-529074 | 0.219110776 | 2.41182E-07 | positive |
| POLD4 | olaparib | 0.219023442 | 7.43402E-09 | positive |
| POLD4 | topotecan | 0.21898339 | 2.0784E-09 | positive |
| POLD4 | KW-2449 | 0.216368264 | 5.12108E-09 | positive |
| POLD4 | cytarabine hydrochloride | 0.216132328 | 4.248E-09 | positive |
| POLD4 | GMX-1778 | 0.214079403 | 2.46721E-08 | positive |
| POLD4 | ouabain | 0.21267991 | 1.00035E-08 | positive |
| POLD4 | PRIMA-1 | 0.211073557 | 3.78684E-08 | positive |
| POLD4 | gossypol | 0.210767564 | 5.99915E-08 | positive |
| POLD4 | LRRK2-IN-1 | 0.21026409 | 2.38852E-06 | positive |
| POLD4 | gemcitabine | 0.208901079 | 7.73931E-08 | positive |

| | | | | |
|---|---|---|---|---|
| POLD4 | GW-843682X | 0.208744656 | 9.27996E-08 | positive |
| POLD4 | linifanib | 0.208309897 | 2.77237E-08 | positive |
| POLD4 | pifithrin-mu | 0.208089605 | 4.5936E-08 | positive |
| POLD4 | TW-37 | 0.207495001 | 1.02661E-07 | positive |
| POLD4 | triptolide | 0.206867626 | 5.1759E-08 | positive |
| POLD4 | OSI-930 | 0.206716827 | 9.11056E-07 | positive |
| POLD4 | SR-II-138A | 0.20654347 | 1.34642E-08 | positive |
| POLD4 | alisertib | 0.205182203 | 4.52844E-08 | positive |
| POLD4 | CAY10618 | 0.204179918 | 5.53104E-08 | positive |
| POLD4 | BMS-345541 | 0.202936286 | 1.10592E-07 | positive |
| POLD4 | Ko-143 | 0.202701872 | 1.38477E-07 | positive |
| POLD4 | SN-38 | 0.202662992 | 1.21291E-06 | positive |
| POLD4 | docetaxel | 0.201521294 | 0.000456058 | positive |
| POLD4 | alvocidib | 0.201498205 | 0.000343768 | positive |
| POLD4 | BRD-K88742110 | 0.201463175 | 3.44382E-07 | positive |
| POLD4 | serdemetan | 0.200675191 | 2.70324E-07 | positive |
| POLD4 | BIX-01294 | 0.200040853 | 1.20705E-07 | positive |
| POLD4 | SID 26681509 | 0.1984866 | 2.26471E-06 | positive |
| POLD4 | JQ-1 | 0.197605758 | 9.81513E-08 | positive |
| POLD4 | SMER-3 | 0.196307761 | 3.78531E-06 | positive |
| POLD4 | CR-1-31B | 0.194816162 | 1.18119E-07 | positive |
| POLD4 | PF-750 | 0.193506156 | 0.000010752 | positive |
| POLD4 | BRD-K28456706 | 0.192653301 | 1.12077E-06 | positive |
| POLD4 | methylstat | 0.191902382 | 0.0000324 | positive |
| POLD4 | marinopyrrole A | 0.191310262 | 0.000091557 | positive |
| POLD4 | CIL55A | 0.191078957 | 0.000846847 | positive |
| POLD4 | BRD-K35604418 | 0.190732326 | 7.02497E-07 | positive |
| POLD4 | PHA-793887 | 0.189268151 | 3.17441E-07 | positive |
| POLD4 | PF-184 | 0.189075887 | 7.69912E-07 | positive |
| POLD4 | nutlin-3 | 0.188568741 | 1.13017E-06 | positive |
| POLD4 | bardoxolone methyl | 0.186473579 | 4.54805E-06 | positive |
| POLD4 | isoliquiritigenin | 0.186390086 | 0.025138063 | positive |
| POLD4 | chlorambucil | 0.186277756 | 9.4747E-07 | positive |
| POLD4 | Compound 23 citrate | 0.185367648 | 1.38821E-06 | positive |
| POLD4 | CCT036477 | 0.185304578 | 1.01771E-06 | positive |
| POLD4 | valdecoxib | 0.184597618 | 2.237E-06 | positive |
| POLD4 | BRD-K66532283 | 0.184311978 | 2.41489E-06 | positive |
| POLD4 | PF-3758309 | 0.183516798 | 0.000871358 | positive |
| POLD4 | SKI-II | 0.181804424 | 0.00001005 | positive |
| POLD4 | NVP-BSK805 | 0.180467759 | 2.53261E-06 | positive |
| POLD4 | crizotinib | 0.180402996 | 2.03015E-06 | positive |
| POLD4 | bortezomib | 0.179903467 | 3.04126E-06 | positive |
| POLD4 | spautin-1 | 0.179654462 | 0.000207821 | positive |
| POLD4 | obatoclax | 0.179345768 | 1.84558E-06 | positive |
| POLD4 | QW-BI-011 | 0.179057065 | 0.037055374 | positive |
| POLD4 | PI-103 | 0.17894114 | 5.51606E-06 | positive |

| | | | | |
|---|---|---|---|---|
| POLD4 | foretinib | 0.177691553 | 7.12591E-06 | positive |
| POLD4 | pevonedistat | 0.176989748 | 4.72909E-06 | positive |
| POLD4 | BRD-K92856060 | 0.176978843 | 8.25759E-06 | positive |
| POLD4 | DBeQ | 0.175160476 | 0.000020252 | positive |
| POLD4 | fingolimod | 0.173372691 | 6.82888E-06 | positive |
| POLD4 | BRD-K29313308 | 0.17297489 | 0.000018448 | positive |
| POLD4 | austocystin D | 0.171508132 | 0.000056433 | positive |
| POLD4 | BRD-K61166597 | 0.171067889 | 0.000012175 | positive |
| POLD4 | ML239 | 0.170958244 | 0.000015947 | positive |
| POLD4 | MLN2238 | 0.170575571 | 8.21658E-06 | positive |
| POLD4 | pazopanib | 0.170175309 | 9.74638E-06 | positive |
| POLD4 | BMS-754807 | 0.169917897 | 0.00003 | positive |
| POLD4 | ABT-737 | 0.169872598 | 0.000043833 | positive |
| POLD4 | prochlorperazine | 0.168990484 | 0.000047319 | positive |
| POLD4 | BRD-K80183349 | 0.168789794 | 0.0000113 | positive |
| POLD4 | R428 | 0.168517208 | 0.000426166 | positive |
| POLD4 | tubastatin A | 0.168399095 | 0.00377128 | positive |
| POLD4 | navitoclax | 0.168049423 | 0.000023745 | positive |
| POLD4 | neuronal differentiation inducer | 0.167299564 | 9.95946E-06 | positive |
| POLD4 | ML050 | 0.166080477 | 0.000041764 | positive |
| POLD4 | CIL41 | 0.166047265 | 0.009732213 | positive |
| POLD4 | BRD-K51490254 | 0.165810111 | 0.000051899 | positive |
| POLD4 | Ki8751 | 0.164870087 | 0.000052412 | positive |
| POLD4 | GW-405833 | 0.164595711 | 0.000014415 | positive |
| POLD4 | CIL70 | 0.163924149 | 0.003090749 | positive |
| POLD4 | BRD-K13999467 | 0.163526316 | 0.000056157 | positive |
| POLD4 | SB-525334 | 0.163448127 | 0.000268398 | positive |
| POLD4 | sorafenib | 0.163410956 | 0.000076542 | positive |
| POLD4 | Merck60 | 0.162978987 | 0.000013514 | positive |
| POLD4 | clofarabine | 0.162493102 | 0.000011471 | positive |
| POLD4 | Ch-55 | 0.162131777 | 0.000158732 | positive |
| POLD4 | HLI 373 | 0.161976134 | 0.000044266 | positive |
| POLD4 | BRD-K41597374 | 0.161929199 | 0.000029472 | positive |
| POLD4 | ML031 | 0.160802741 | 0.000121551 | positive |
| POLD4 | Mdivi-1 | 0.159768226 | 0.000068844 | positive |
| POLD4 | brefeldin A | 0.159468449 | 0.00014799 | positive |
| POLD4 | NSC19630 | 0.158145147 | 0.00071512 | positive |
| POLD4 | PDMP | 0.156594797 | 0.000118929 | positive |
| POLD4 | NSC48300 | 0.155339018 | 0.000045444 | positive |
| POLD4 | tipifarnib-P1 | 0.154826729 | 0.000064816 | positive |
| POLD4 | SNS-032 | 0.154437638 | 0.000124088 | positive |
| POLD4 | MG-132 | 0.154073258 | 0.043220412 | positive |
| POLD4 | AZD7545 | 0.153826221 | 0.000174724 | positive |
| POLD4 | SU11274 | 0.15212892 | 0.000111083 | positive |
| POLD4 | sotrastaurin | 0.151673202 | 0.000954699 | positive |
| POLD4 | NVP-BEZ235 | 0.149587196 | 0.001934637 | positive |

| | | | | |
|---|---|---|---|---|
| POLD4 | AT7867 | 0.147482351 | 0.000183721 | positive |
| POLD4 | UNC0638 | 0.146528547 | 0.000299689 | positive |
| POLD4 | tretinoin | 0.145884426 | 0.000240288 | positive |
| POLD4 | darinaparsin | 0.14549919 | 0.015890207 | positive |
| POLD4 | N9-isopropylolomoucine | 0.14545346 | 0.000262129 | positive |
| POLD4 | BRD-K24690302 | 0.1447697 | 0.000363021 | positive |
| POLD4 | AZ-3146 | 0.144334812 | 0.000314203 | positive |
| POLD4 | I-BET151 | 0.144129336 | 0.000111128 | positive |
| POLD4 | 16-beta-bromoandrosterone | 0.143148106 | 0.001573692 | positive |
| POLD4 | AZD1480 | 0.142833343 | 0.031770706 | positive |
| POLD4 | BRD-A86708339 | 0.142703305 | 0.01562858 | positive |
| POLD4 | temsirolimus | 0.142050262 | 0.016354192 | positive |
| POLD4 | RITA | 0.141649611 | 0.000414856 | positive |
| POLD4 | BRD-K85133207 | 0.14118216 | 0.000818431 | positive |
| POLD4 | masitinib | 0.141038583 | 0.000441115 | positive |
| POLD4 | BRD6340 | 0.140558822 | 0.000328123 | positive |
| POLD4 | HBX-41108 | 0.14054187 | 0.00605358 | positive |
| POLD4 | methotrexate | 0.140004135 | 0.000536968 | positive |
| POLD4 | regorafenib | 0.138449521 | 0.002653643 | positive |
| POLD4 | StemRegenin 1 | 0.137615646 | 0.000467327 | positive |
| POLD4 | BRD-K11533227 | 0.137310941 | 0.001085411 | positive |
| POLD4 | BRD-K45681478 | 0.137213506 | 0.001191012 | positive |
| POLD4 | AZD4547 | 0.134353109 | 0.001978693 | positive |
| POLD4 | avrainvillamide | 0.133802366 | 0.004835864 | positive |
| POLD4 | BMS-195614 | 0.132665363 | 0.01679602 | positive |
| POLD4 | PF-573228 | 0.131953224 | 0.000677731 | positive |
| POLD4 | linsitinib | 0.131372529 | 0.001449963 | positive |
| POLD4 | tacrolimus | 0.129644091 | 0.001624412 | positive |
| POLD4 | NVP-ADW742 | 0.129112619 | 0.002047301 | positive |
| POLD4 | itraconazole | 0.129101447 | 0.035494173 | positive |
| POLD4 | TPCA-1 | 0.12852704 | 0.000896546 | positive |
| POLD4 | zebularine | 0.128358973 | 0.001033 | positive |
| POLD4 | BRD4132 | 0.128007875 | 0.031433085 | positive |
| POLD4 | tandutinib | 0.12698931 | 0.002626147 | positive |
| POLD4 | COL-3 | 0.126756336 | 0.005331382 | positive |
| POLD4 | BIBR-1532 | 0.126422643 | 0.003136965 | positive |
| POLD4 | SNX-2112 | 0.126347777 | 0.000733892 | positive |
| POLD4 | KU-60019 | 0.124155177 | 0.001391222 | positive |
| POLD4 | BRD1835 | 0.123319289 | 0.00607636 | positive |
| POLD4 | RG-108 | 0.121801909 | 0.003623293 | positive |
| POLD4 | purmorphamine | 0.120902582 | 0.00768868 | positive |
| POLD4 | NVP-TAE684 | 0.12020088 | 0.005818787 | positive |
| POLD4 | BRD9876 | 0.119995459 | 0.031472155 | positive |
| POLD4 | lenvatinib | 0.119278074 | 0.004368837 | positive |
| POLD4 | AT13387 | 0.119167255 | 0.041943152 | positive |
| POLD4 | fluorouracil | 0.118502084 | 0.002069743 | positive |

| | | | | |
|---|---|---|---|---|
| POLD4 | KU-0063794 | 0.117730396 | 0.003348844 | positive |
| POLD4 | brivanib | 0.117566196 | 0.003283985 | positive |
| POLD4 | quizartinib | 0.117036699 | 0.025706847 | positive |
| POLD4 | BRD-A02303741 | 0.116938257 | 0.018939964 | positive |
| POLD4 | elocalcitol | 0.115730495 | 0.002879253 | positive |
| POLD4 | trifluoperazine | 0.115557517 | 0.0130947 | positive |
| POLD4 | CD-1530 | 0.114611141 | 0.023493767 | positive |
| POLD4 | LE-135 | 0.113670046 | 0.00721297 | positive |
| POLD4 | decitabine | 0.113281369 | 0.0027152 | positive |
| POLD4 | epigallocatechin-3-monogallate | 0.112190293 | 0.010095639 | positive |
| POLD4 | Compound 1541A | 0.111856356 | 0.036718221 | positive |
| POLD4 | sunitinib | 0.110788074 | 0.005303924 | positive |
| POLD4 | WZ8040 | 0.110510105 | 0.020428218 | positive |
| POLD4 | ML029 | 0.109549997 | 0.010439583 | positive |
| POLD4 | GSK525762A | 0.106958201 | 0.004937781 | positive |
| POLD4 | RAF265 | 0.106192362 | 0.038178795 | positive |
| POLD4 | XL765 | 0.105854706 | 0.038032129 | positive |
| POLD4 | BRD-K55116708 | 0.105381315 | 0.014854501 | positive |
| POLD4 | TG-101348 | 0.103075806 | 0.010109378 | positive |
| POLD4 | OSI-027 | 0.102102795 | 0.013687826 | positive |
| POLD4 | bendamustine | 0.101716063 | 0.016568755 | positive |
| POLD4 | AZD8055 | 0.101295761 | 0.011144126 | positive |
| POLD4 | MGCD-265 | 0.101271343 | 0.009490749 | positive |
| POLD4 | cytochalasin B | 0.101198842 | 0.032923936 | positive |
| POLD4 | PIK-93 | 0.099831354 | 0.015029596 | positive |
| POLD4 | erastin | 0.099809356 | 0.01611085 | positive |
| POLD4 | BRD-A71883111 | 0.099669433 | 0.018043678 | positive |
| POLD4 | sirolimus | 0.098178548 | 0.01276858 | positive |
| POLD4 | momelotinib | 0.097795518 | 0.016292846 | positive |
| POLD4 | AA-COCF3 | 0.096237886 | 0.020146011 | positive |
| POLD4 | BMS-270394 | 0.095804184 | 0.029231371 | positive |
| POLD4 | tosedostat | 0.095314661 | 0.023869914 | positive |
| POLD4 | cabozantinib | 0.094607262 | 0.037606156 | positive |
| POLD4 | AZD7762 | 0.093008097 | 0.016625905 | positive |
| POLD4 | PF-543 | 0.092036741 | 0.040816767 | positive |
| POLD4 | PD318088 | -0.088720368 | 0.048295994 | negative |
| POLD4 | selumetinib | -0.090806996 | 0.047929365 | negative |
| POLD4 | pluripotin | -0.095286019 | 0.036530285 | negative |
| POLD4 | vandetanib | -0.113936936 | 0.013200464 | negative |
| POLD4 | IC-87114 | -0.138516278 | 0.007050186 | negative |
| POLD4 | trametinib | -0.151296539 | 0.024537313 | negative |
| POLD4 | saracatinib | -0.191513496 | 2.5395E-06 | negative |
| POLD4 | dasatinib | -0.218715051 | 3.78387E-08 | negative |
| POLD4 | BRD-K30748066 | 0.294424079 | 0.074387524 | not significant |
| POLD4 | tozasertib | 0.220219331 | 0.172644242 | not significant |
| POLD4 | BRD-K34099515 | 0.212781353 | 0.641592415 | not significant |

| | | | | |
|---|---|---|---|---|
| POLD4 | BRD-K09344309 | 0.195528187 | 0.505697486 | not significant |
| POLD4 | BRD-K48334597 | 0.191508197 | 0.060798488 | not significant |
| POLD4 | BRD-K30019337 | 0.159746651 | 0.458361003 | not significant |
| POLD4 | cyanoquinoline 11 | 0.14727844 | 0.056068804 | not significant |
| POLD4 | BRD-K75293299 | 0.144507236 | 0.503931554 | not significant |
| POLD4 | 968 | 0.139393929 | 0.899628325 | not significant |
| POLD4 | cimetidine | 0.138786198 | 0.904322701 | not significant |
| POLD4 | BRD-K29086754 | 0.137866888 | 0.414716306 | not significant |
| POLD4 | SR8278 | 0.134042222 | 0.687150393 | not significant |
| POLD4 | BRD-M00053801 | 0.132957939 | 0.114027303 | not significant |
| POLD4 | PRIMA-1-Met | 0.127403923 | 0.052838476 | not significant |
| POLD4 | silmitasertib | 0.119168575 | 0.116887468 | not significant |
| POLD4 | BRD-K84807411 | 0.117102276 | 0.632743695 | not significant |
| POLD4 | MI-2 | 0.116958661 | 0.073968215 | not significant |
| POLD4 | CIL55 | 0.113914569 | 0.594978301 | not significant |
| POLD4 | AT-406 | 0.110589668 | 0.22718038 | not significant |
| POLD4 | skepinone-L | 0.106170132 | 0.087603303 | not significant |
| POLD4 | fulvestrant | 0.102366704 | 0.764018765 | not significant |
| POLD4 | ABT-199 | 0.101909115 | 0.062761888 | not significant |
| POLD4 | QS-11 | 0.100600855 | 0.679154518 | not significant |
| POLD4 | CAY10594 | 0.100535905 | 0.061576365 | not significant |
| POLD4 | ETP-46464 | 0.0966418 | 0.064568952 | not significant |
| POLD4 | BRD-K27986637 | 0.096623693 | 0.519523346 | not significant |
| POLD4 | SR1001 | 0.094482356 | 0.134282373 | not significant |
| POLD4 | parthenolide | 0.09311457 | 0.078809071 | not significant |
| POLD4 | AM-580 | 0.092556859 | 0.054777551 | not significant |
| POLD4 | bafilomycin A1 | 0.091415526 | 0.876370175 | not significant |
| POLD4 | nintedanib | 0.090270645 | 0.069669239 | not significant |
| POLD4 | BRD9647 | 0.089870475 | 0.060916981 | not significant |
| POLD4 | NSC 74859 | 0.089639666 | 0.067445317 | not significant |
| POLD4 | staurosporine | 0.088094915 | 0.497504171 | not significant |
| POLD4 | vorapaxar | 0.086331977 | 0.057099225 | not significant |
| POLD4 | tivozanib | 0.085326607 | 0.065203705 | not significant |
| POLD4 | BRD-K96970199 | 0.084645679 | 0.998763577 | not significant |
| POLD4 | BIRB-796 | 0.083826955 | 0.06104497 | not significant |
| POLD4 | PYR-41 | 0.082497202 | 0.107288899 | not significant |
| POLD4 | nilotinib | 0.080718363 | 0.25036826 | not significant |
| POLD4 | IU1 | 0.080425025 | 0.325134671 | not significant |
| POLD4 | KU-55933 | 0.078900294 | 0.066658825 | not significant |
| POLD4 | importazole | 0.078276353 | 0.178608475 | not significant |
| POLD4 | NPC-26 | 0.076269076 | 0.44393561 | not significant |
| POLD4 | FSC231 | 0.075966238 | 0.999449861 | not significant |
| POLD4 | YM-155 | 0.075565332 | 0.110266832 | not significant |
| POLD4 | BRD-K52037352 | 0.073973979 | 0.232446828 | not significant |
| POLD4 | BRD-K78574327 | 0.073649943 | 0.969647507 | not significant |
| POLD4 | BRD-K50799972 | 0.072967469 | 0.266284893 | not significant |

| | | | | |
|---|---|---|---|---|
| POLD4 | BRD-K90370028 | 0.07273683 | 0.854738894 | not significant |
| POLD4 | JW-480 | 0.072191912 | 0.678430459 | not significant |
| POLD4 | tigecycline | 0.071812573 | 0.248878368 | not significant |
| POLD4 | fluvastatin | 0.071526252 | 0.159185791 | not significant |
| POLD4 | necrostatin-1 | 0.071449188 | 0.125463596 | not significant |
| POLD4 | isonicotinohydroxamic acid | 0.068478319 | 0.957649701 | not significant |
| POLD4 | GDC-0941 | 0.068250382 | 0.129090421 | not significant |
| POLD4 | BRD-K02251932 | 0.068118283 | 0.175929818 | not significant |
| POLD4 | MK-2206 | 0.067301656 | 0.124448027 | not significant |
| POLD4 | GSK-3 inhibitor IX | 0.066627426 | 0.122681013 | not significant |
| POLD4 | BRD-K86535717 | 0.066289985 | 0.840704188 | not significant |
| POLD4 | NSC23766 | 0.066093154 | 0.111016977 | not significant |
| POLD4 | necrostatin-7 | 0.066064948 | 0.146370506 | not significant |
| POLD4 | salermide | 0.065200198 | 0.485392033 | not significant |
| POLD4 | KU 0060648 | 0.064391233 | 0.121036958 | not significant |
| POLD4 | ML162 | 0.063841245 | 0.134289343 | not significant |
| POLD4 | bexarotene | 0.06332532 | 0.14921379 | not significant |
| POLD4 | dexamethasone | 0.063143165 | 0.141517162 | not significant |
| POLD4 | dacarbazine | 0.062657219 | 0.116869483 | not significant |
| POLD4 | ML006 | 0.062466625 | 0.233742603 | not significant |
| POLD4 | BRD-K19103580 | 0.061951668 | 0.210925568 | not significant |
| POLD4 | C6-ceramide | 0.06144911 | 0.550708784 | not significant |
| POLD4 | pandacostat | 0.059986049 | 0.198889404 | not significant |
| POLD4 | GSK4112 | 0.059802305 | 0.224178064 | not significant |
| POLD4 | tanespimycin | 0.059602921 | 0.25367929 | not significant |
| POLD4 | BRD-K96431673 | 0.057685798 | 0.986199957 | not significant |
| POLD4 | BRD-K42260513 | 0.057493576 | 0.991730811 | not significant |
| POLD4 | imatinib | 0.056713756 | 0.194169477 | not significant |
| POLD4 | FGIN-1-27 | 0.056534742 | 0.686077906 | not significant |
| POLD4 | BRD-K63431240 | 0.056517891 | 0.204306323 | not significant |
| POLD4 | JW-55 | 0.056378064 | 0.680523285 | not significant |
| POLD4 | PRL-3 inhibitor I | 0.054837781 | 0.654581252 | not significant |
| POLD4 | BRD-K49290616 | 0.054558907 | 0.902615266 | not significant |
| POLD4 | BYL-719 | 0.054016471 | 0.438098813 | not significant |
| POLD4 | WZ4002 | 0.052392985 | 0.472564417 | not significant |
| POLD4 | ruxolitinib | 0.051124497 | 0.243397941 | not significant |
| POLD4 | SRT-1720 | 0.05053517 | 0.279327796 | not significant |
| POLD4 | EX-527 | 0.050506356 | 0.66730153 | not significant |
| POLD4 | GANT-61 | 0.048925554 | 0.416719545 | not significant |
| POLD4 | ZSTK474 | 0.048334745 | 0.381265714 | not significant |
| POLD4 | ML203 | 0.048227964 | 0.364109133 | not significant |
| POLD4 | ML258 | 0.047627053 | 0.655025546 | not significant |
| POLD4 | pitstop2 | 0.046772992 | 0.815614337 | not significant |
| POLD4 | blebbistatin | 0.046426211 | 0.953628268 | not significant |
| POLD4 | BRD-K27224038 | 0.046053316 | 0.878212418 | not significant |
| POLD4 | A-804598 | 0.045577611 | 0.784323593 | not significant |

| | | | | |
|---|---|---|---|---|
| POLD4 | ML210 | 0.04473189 | 0.300444597 | not significant |
| POLD4 | MLN2480 | 0.044533936 | 0.698048694 | not significant |
| POLD4 | AC55649 | 0.040717556 | 0.747131533 | not significant |
| POLD4 | ML312 | 0.040620934 | 0.983011224 | not significant |
| POLD4 | ciclosporin | 0.040554828 | 0.481143639 | not significant |
| POLD4 | BRD-K71781559 | 0.040385473 | 0.988193932 | not significant |
| POLD4 | BRD-K41334119 | 0.039660691 | 0.955694993 | not significant |
| POLD4 | veliparib | 0.038994117 | 0.640529655 | not significant |
| POLD4 | L-685458 | 0.037835794 | 0.558128467 | not significant |
| POLD4 | pifithrin-alpha | 0.037578437 | 0.567467371 | not significant |
| POLD4 | lomeguatrib | 0.036398912 | 0.540447256 | not significant |
| POLD4 | BRD-K14844214 | 0.036190996 | 0.645722787 | not significant |
| POLD4 | VER-155008 | 0.035890764 | 0.424942837 | not significant |
| POLD4 | WAY-362450 | 0.035322977 | 0.99852118 | not significant |
| POLD4 | nelarabine | 0.035166915 | 0.895118774 | not significant |
| POLD4 | CHIR-99021 | 0.034767006 | 0.447386409 | not significant |
| POLD4 | pyrazolanthrone | 0.034394708 | 0.526787625 | not significant |
| POLD4 | JW-74 | 0.034174071 | 0.729346314 | not significant |
| POLD4 | NSC30930 | 0.033210762 | 0.671077022 | not significant |
| POLD4 | simvastatin | 0.031689147 | 0.624279813 | not significant |
| POLD4 | thalidomide | 0.030819969 | 0.899087698 | not significant |
| POLD4 | CBB-1007 | 0.029535286 | 0.888578212 | not significant |
| POLD4 | 1S,3R-RSL-3 | 0.029515955 | 0.504421321 | not significant |
| POLD4 | bleomycin A2 | 0.029393584 | 0.638144511 | not significant |
| POLD4 | HC-067047 | 0.029273452 | 0.670597739 | not significant |
| POLD4 | BRD-K51831558 | 0.029115195 | 0.716290147 | not significant |
| POLD4 | hyperforin | 0.02869306 | 0.780813147 | not significant |
| POLD4 | AGK-2 | 0.027907093 | 0.948122459 | not significant |
| POLD4 | ML320 | 0.027497143 | 0.599286771 | not significant |
| POLD4 | CI-976 | 0.027253768 | 0.873017117 | not significant |
| POLD4 | IPR-456 | 0.025682632 | 0.88526284 | not significant |
| POLD4 | CIL56 | 0.025491141 | 0.706960029 | not significant |
| POLD4 | etomoxir | 0.025104418 | 0.971372384 | not significant |
| POLD4 | bosutinib | 0.023847631 | 0.60035224 | not significant |
| POLD4 | BMS-536924 | 0.023175357 | 0.712982149 | not significant |
| POLD4 | SZ4TA2 | 0.021775808 | 0.886316517 | not significant |
| POLD4 | BRD8958 | 0.021042668 | 0.982653634 | not significant |
| POLD4 | myriocin | 0.019621016 | 0.97336935 | not significant |
| POLD4 | lovastatin | 0.017322224 | 0.776878293 | not significant |
| POLD4 | tamoxifen | 0.017193134 | 0.837608592 | not significant |
| POLD4 | azacitidine | 0.017133861 | 0.754876986 | not significant |
| POLD4 | GSK1059615 | 0.016588024 | 0.964940693 | not significant |
| POLD4 | BRD1378 | 0.014954843 | 0.921071047 | not significant |
| POLD4 | BRD-K17060750 | 0.014639407 | 0.809950885 | not significant |
| POLD4 | palmostatin B | 0.014530275 | 0.92442477 | not significant |
| POLD4 | TG-100-115 | 0.013694922 | 0.833565057 | not significant |

| | | | | |
|---|---|---|---|---|
| POLD4 | BRD8899 | 0.013597379 | 0.91981573 | not significant |
| POLD4 | BRD-K37390332 | 0.012759447 | 0.977051675 | not significant |
| POLD4 | BRD-K09587429 | 0.011343523 | 0.887864772 | not significant |
| POLD4 | ML334 diastereomer | 0.011081155 | 0.911086899 | not significant |
| POLD4 | canertinib | 0.009734798 | 0.865752203 | not significant |
| POLD4 | neratinib | 0.008061943 | 0.881855112 | not significant |
| POLD4 | istradefylline | 0.007944013 | 0.971848343 | not significant |
| POLD4 | lapatinib | 0.007165316 | 0.894782805 | not significant |
| POLD4 | BRD5468 | 0.007088584 | 0.955740626 | not significant |
| POLD4 | cediranib | 0.007057187 | 0.970273659 | not significant |
| POLD4 | KH-CB19 | 0.006842743 | 0.959242626 | not significant |
| POLD4 | BRD-K04800985 | 0.005326863 | 0.99978544 | not significant |
| POLD4 | MI-1 | 0.003420893 | 0.979806831 | not significant |
| POLD4 | VU0155056 | 0.003100183 | 0.996485324 | not significant |
| POLD4 | GDC-0879 | 0.003019557 | 0.968484108 | not significant |
| POLD4 | vemurafenib | 0.000732431 | 0.993269869 | not significant |
| POLD4 | BRD-K16147474 | 0.000067311 | 0.999284202 | not significant |
| POLD4 | DNMDP | -0.001578849 | 0.988183986 | not significant |
| POLD4 | CAY10576 | -0.003292147 | 0.973967155 | not significant |
| POLD4 | erismodegib | -0.004598305 | 0.970811783 | not significant |
| POLD4 | sitagliptin | -0.007301732 | 0.997718377 | not significant |
| POLD4 | BRD-K02492147 | -0.007395515 | 0.92388828 | not significant |
| POLD4 | GSK2636771 | -0.007822117 | 0.956092521 | not significant |
| POLD4 | BRD-K34485477 | -0.010944118 | 0.998562234 | not significant |
| POLD4 | BRD-K64610608 | -0.01101608 | 0.944840731 | not significant |
| POLD4 | RO4929097 | -0.013368009 | 0.883546442 | not significant |
| POLD4 | betulinic acid | -0.014374062 | 0.948096759 | not significant |
| POLD4 | sildenafil | -0.015023517 | 0.930393447 | not significant |
| POLD4 | temozolomide | -0.01661769 | 0.83664571 | not significant |
| POLD4 | AZD6482 | -0.017601685 | 0.808678943 | not significant |
| POLD4 | myricetin | -0.017744242 | 0.897887063 | not significant |
| POLD4 | BRD-K99006945 | -0.017888196 | 0.830977631 | not significant |
| POLD4 | SGX-523 | -0.018100253 | 0.808627695 | not significant |
| POLD4 | BRD-K44224150 | -0.019263888 | 0.996932862 | not significant |
| POLD4 | BRD-K33514849 | -0.019275725 | 0.999288401 | not significant |
| POLD4 | SB-431542 | -0.019332082 | 0.878633305 | not significant |
| POLD4 | PLX-4720 | -0.021749628 | 0.808145895 | not significant |
| POLD4 | BEC | -0.022136001 | 0.999979174 | not significant |
| POLD4 | tamatinib | -0.023377052 | 0.807532432 | not significant |
| POLD4 | CID-5951923 | -0.026951515 | 0.785697913 | not significant |
| POLD4 | BRD-K33199242 | -0.027870259 | 0.965292006 | not significant |
| POLD4 | O-6-benzylguanine | -0.032762358 | 0.825561643 | not significant |
| POLD4 | afatinib | -0.033627328 | 0.515625019 | not significant |
| POLD4 | UNC0321 | -0.035873147 | 0.764759626 | not significant |
| POLD4 | avicin D | -0.036151603 | 0.730777231 | not significant |
| POLD4 | ifosfamide | -0.036325142 | 0.874454436 | not significant |

| | | | | |
|---|---|---|---|---|
| POLD4 | SJ-172550 | -0.036670793 | 0.760852742 | not significant |
| POLD4 | semagacestat | -0.039762328 | 0.77988939 | not significant |
| POLD4 | gefitinib | -0.042482576 | 0.436995201 | not significant |
| POLD4 | fumonisin B1 | -0.047899555 | 0.424067019 | not significant |
| POLD4 | ibrutinib | -0.050847317 | 0.40562591 | not significant |
| POLD4 | PF-4800567 hydrochloride | -0.05156955 | 0.999586205 | not significant |
| POLD4 | BCL-LZH-4 | -0.057193092 | 0.788453092 | not significant |
| POLD4 | MK-0752 | -0.058834887 | 0.407555325 | not significant |
| POLD4 | cyclophosphamide | -0.059689632 | 0.993392039 | not significant |
| POLD4 | ML083 | -0.06054968 | 0.592590981 | not significant |
| POLD4 | VAF-347 | -0.06086336 | 0.343630231 | not significant |
| POLD4 | PD 153035 | -0.06370091 | 0.280430886 | not significant |
| POLD4 | erlotinib | -0.064774874 | 0.152896617 | not significant |
| POLD4 | dabrafenib | -0.066525554 | 0.389445861 | not significant |
| POLD4 | BRD-K48477130 | -0.067742858 | 0.733743548 | not significant |
| POLD4 | birinapant | -0.071727305 | 0.23229598 | not significant |
| POLD4 | TGX-221 | -0.083287484 | 0.178442263 | not significant |
| POLD4 | CAL-101 | -0.084214331 | 0.116899059 | not significant |
| POLD4 | procarbazine | -0.086710135 | 0.554171601 | not significant |
| POLD4 | BRD-A05715709 | -0.097658473 | 0.497572193 | not significant |
| POLD4 | abiraterone | -0.100742997 | 0.451216829 | not significant |
| POLD4 | LY-2157299 | -0.154842456 | 0.428597919 | not significant |
